# Development and Characterization of Novel Genic-SSR Markers in Apple-Juniper Rust Pathogen *Gymnosporangium yamadae* (Pucciniales: Pucciniaceae) Using Next-Generation Sequencing

**DOI:** 10.3390/ijms19041178

**Published:** 2018-04-12

**Authors:** Si-Qi Tao, Bin Cao, Cheng-Ming Tian, Ying-Mei Liang

**Affiliations:** 1 The Key Laboratory for Silviculture and Conservation of Ministry of Education, Beijing Forestry University, Beijing 100083, China; taosq_bjfu@163.com (S.-Q.T.); 398031987@163.com (B.C.); chengmt@bjfu.edu.cn (C.-M.T.); 2 Museum of Beijing Forestry University, Beijing Forestry University, Beijing 100083, China

**Keywords:** Apple-Juniper rust, *Gymnosporangium yamadae*, simple sequence repeat, next-generation sequencing

## Abstract

The Apple-Juniper rust, *Gymnosporangium yamadae*, is an economically important pathogen of apples and junipers in Asia. The absence of markers has hampered the study of the genetic diversity of this widespread pathogen. In our study, we developed twenty-two novel microsatellite markers for *G. yamadae* from randomly sequenced regions of the transcriptome, using next-generation sequencing methods. These polymorphic markers were also tested on 96 *G. yamadae* individuals from two geographical populations. The allele numbers ranged from 2 to 9 with an average value of 6 per locus. The polymorphism information content (PIC) values ranged from 0.099 to 0.782 with an average value of 0.48. Furthermore, the observed (H_O_) and expected (H_E_) heterozygosity ranged from 0.000 to 0.683 and 0.04 to 0.820, respectively. These novel developed microsatellites provide abundant molecular markers for investigating the genetic structure and genetic diversity of *G. yamadae*, which will help us to better understand disease epidemics and the origin and migration routes of the Apple-Juniper rust pathogen. Further studies will also be completed to dissect how human activities influence the formation of current population structures. Furthermore, these SSR (simple sequence repeat) markers can also be used as tools to identify virulence by mapping the whole genomes of different virulent populations. These markers will, thus, assist the development of effective risk-assessment models and management systems for the Apple-Juniper rust pathogen.

## 1. Introduction

The domesticated apple (*Malus* × *domestica* Borkh., family Rosaceae, tribe Pyreae) is the main fruit crop of temperate regions of the world [1]. The production of apples in most areas of northern China accounts for more than half of the world’s output and, thus, plays an essential strategic role in export, agricultural structure adjustment, and farmer incomes [2,3]. However, hectares of apple production can be limited by a variety of diseases caused by fungi [4,5]. *Gymnosporangium yamadae* Miyabe, belonging to the Pucciniaceae family, is a serious rust pathogen of domestic apples as well as cultivated junipers, which causes much concern in Asia [6,7]. As one of the non-European *Gymnosporangium* species, it has been listed as an A1 quarantine organism by European and Mediterranean Plant Protection Organization (EPPO) [8]. In China, this species has a wide range of distribution throughout the country and reduces fruit yield by inhibiting photosynthesis and increasing respiration due to infection, which threatens the economic development of local orchards [9,10,11,12]. Under natural conditions, this pathogen is believed to be heteroecious (two taxonomically unrelated hosts are needed to complete its life cycle) and macrocyclic (0-spermatia, I-aeciospores, III-teliospores and IV-basidiospores) [6,7]. The telial host of *G. yamadae*, *Juniperus chinensis* L., exists commonly in parks, roadsides and graveyards, and the alternate aecial hosts, *Malus baccata*, *M. domestica*, *M. prunifolia* and *M. toringo* and other several species of *Malus*, are often planted in the same sites as *J. chinensis*. With the increased movement of junipers as nursery stock and cultivated apple as favored fruits for consumption, the risk of disease occurrence is rather high [13]. Although the primary method of rust control is the application of protective or systemic fungicides and blocking of the infection cycle, the most popular method for disease control is the use of resistant varieties, due to its efficiency, low cost and reduced impact on the environment [14].

Rust fungi are obligate parasites that only infect specific plant genera or species [7], it shows genetic diversity [15] and there are many studies focused on the ability of rust fungi to adapt. There has been a long history of breeders fighting against the appearance of new rust races that have overcome resistance genes in crops and trees [16,17,18,19]. Twenty *Malus* species have been tested by inoculation experiments in 1984 and the results indicated that only *Malus halliana* Koehne showed high resistance to infection by basidiospores of *G. yamadae* [20]. However, the genetic diversity, population structure and gene flow of the *G. yamadae* population remained unclear due to the lack of research on molecular markers, which hindered the effective prevention and control of this species based on genomic information.

For the wheat stem rust pathogen, *Puccinia graminis* f. sp. *tritici,* and wheat stripe rust pathogen, *P. striiformis* f. sp. *tritici*, the population structure has been studied using molecular markers since the 1980s [21,22,23]. These markers proved useful in studying genetic diversity; however, each has limitations in application, such as the issue of replicability, ambiguous fragment size, and homoplasy. Especially, in the case of certain dikaryotic organisms, codominant markers such as simple sequence repeats (SSRs-microsatellite) have been shown to be more informative in revealing genetic variation compared to dominant markers [24,25]. Microsatellites are regions of DNA consisting of short, tandemly repeated motifs (1–6 bp in length), which have a high level of polymorphism and are widely dispersed in the genomes of all prokaryotic and eukaryotic organisms [24,26]. Microsatellite analyses are relatively simple and easier to score than other genetic markers (e.g., restriction fragment length polymorphism (RFLP), random amplified polymorphic DNA (RAPD) and amplified fragment length polymorphism (AFLP) because their gel band patterns are easy to detect, producing unambiguous results.

Thus, microsatellites have recently been developed and validated in many rust fungi, such as *P. striiformis* f. sp. *tritici*, *P. graminis* f. sp. *tritici*, *P. coronata*, *P. novopanici*, *P. triticina* and coffee rust *Hemileia vastatrix* [22,27,28,29,30,31,32,33,34,35]. However, no microsatellite primers specific to *G. yamadae* have been developed to date, so microsatellites for the studies of *G. yamadae* are urgently required. The traditional approaches to microsatellite development, such as massive-scale cloning and sequencing of DNA or EST libraries, are labor intensive and time-consuming. With the rapid development of next-generation sequencing technology, an alternative approach to identify a great number of novel microsatellites based on large-scale RNA-seq became fast, cost-effective and reliable, especially for non-model species [36].

In our study, we used NGS (next generation sequencing) to sequence the *G. yamadae* transcriptome and screened transcript sequences obtained by de novo assembly to detect microsatellites. We then validated the SSR primer sets using *G. yamadae* samples from two geographical areas in China. This is the first time that novel microsatellites for *G. yamadae* have been identified. The developed markers will be important resources to further determine the genetic diversity and genetic structure of this severe pathogen.

## 2. Results

### 2.1. Pair-End Sequences Obtained with the Illumina de Novo Sequencing

In our study, we built the transcriptome of *G. yamadae* during the telial stage of its lifecycle. Three biological replicates were collected, and cDNA libraries were independently constructed and subsequently sequenced using an Illumina Hi-Seq 2000 platform. Approximately 150 million 100-bp paired end (PE) raw reads were generated from each *G. yamadae* cDNA library (Table 1). These data have been deposited in the NCBI Sequence Read Archive under accession numbers SRR5167035–SRR5167040. After removing the adapters and low Phred quality sequences, approximately 150 million high-quality sequence reads were obtained from the three *G. yamadae* libraries (Table 1).

The trimmed reads were then assembled into transcriptomes using the Trinity package [37]; 49,318 transcripts, with a mean length of 1006 bp and an N50 value of 1957, were obtained for *G. yamadae*. Using overlapping information from high-quality reads, we identified 35,102 unigenes for *G. yamadae*, with average lengths of 756 (Table 2).

### 2.2. Mining of Genic SSRs

A total of 35,102 unigenes covering 26,521,164 bp of the *G. yamadae* genome were identified in our data. Of these, 3942 sequences with SSR were detected, and 1136 sequences contained more than one SSR.

### 2.3. Distribution of SSR Motif

The proportions of six different SSR unit sizes were not evenly distributed among all SSRs. Different repeat units occurred at the following frequencies: 37.27% for mono-nucleotides, 34.88% for di-nucleotides, and 23.90% for tri-nucleotides; these accounted for the largest proportions. An additional 2.55%, 0.55%, and 0.87% of the repeats were tetra-nucleotides, penta-nucleotides and hexa-nucleotides, respectively (Table 3). We divided the motif repeat types into seven groups. A repeat number of 5–8 was abundant in the di- and tri-nucleotide motif types, and a repeat number of 9–12 was abundant in the mono-nucleotide motif type. Of the 1884 mono-nucleotide motifs, the (A/T)_n_ mono-nucleotide repeat motif was the most abundant in the dataset. The three other main unit types were (AT/AT)_n_ di-nucleotides, (ACC/GGT)_n_ and (ATC/ATG)_n_ in tri-nucleotides and (AAAT/ATTT)_n_ in tetra-nucleotides, which occurred at frequencies of 15.43%, 4.23%, and 0.26%, respectively (Table 3).

**Figure 1:**
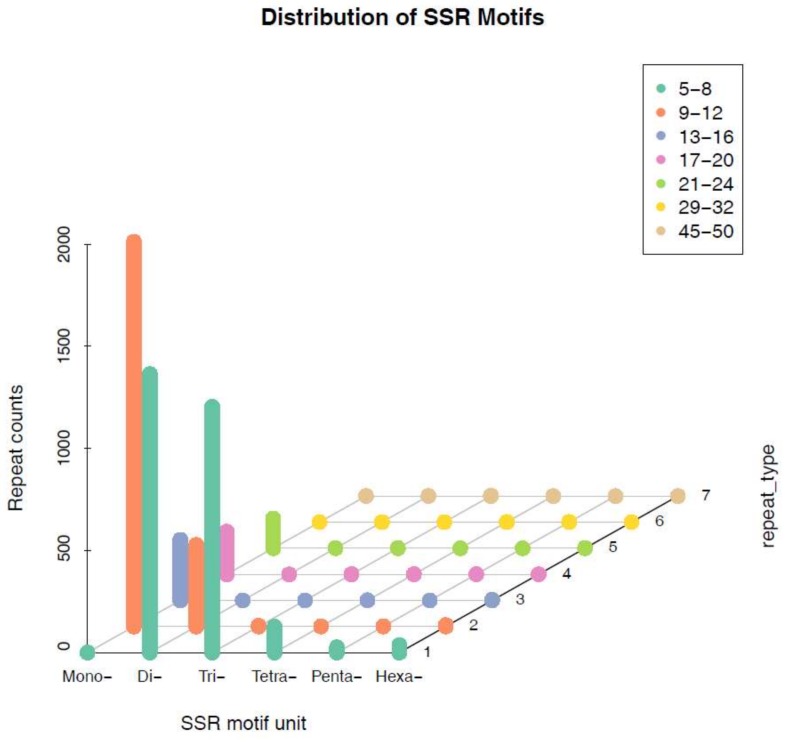
The distribution of SSR motif and repeat numbers.

Further analysis indicated that the copy numbers of different repeat motifs were distributed unevenly (Table 4). The copy numbers of different motif types ranged from 5 to 24, and the four most abundant copy numbers for SSRs were 10 (24.54%), 6 (18.31%), 5 (14.48%), and 7 (10.62%). According to the distribution of microsatellites (Figure 1, Table 4), it seems that the number of loci decrease with the increase in corresponding motif repeats.

### 2.4. PCR Amplification and Polymorphism of Genic-SSRs

Using the software Primer 3 [38], 2832 SSR primers were designed according to the flanking sequences of the 3942 SSR-containing sequences. Of these, 88 primers were randomly selected and synthesized for initial validation on eight individuals. The information about the selected primers is given in Table S1, and the information about the specimens used for amplification is given in Table S2. The PCR amplification results were evaluated by gel electrophoresis. Of the 88 primers, PCR amplicons were not obtained for 40 markers (45%) (Table S3), and the successfully amplified markers used are described in Supplementary Materials. The remaining primers (from previous steps) were tested on twenty individuals (Table S4) of *G. yamadae* using a capillary sequencer, whose forward primers were modified by the PC tail (Primer tail C) (5′ CAGGACCAGGCTACCGTG 3′) to test their genotyping efficiency and marker polymorphism in *G. yamadae*. Among the 48 primers that produced amplicons, 26 (54%) were not polymorphic (Table S5), including eighteen di-nucleotide, four tri-nucleotide, one penta-nucleotide and one hexa-nucleotide repeat markers. The remaining 22 loci have polymorphic amplification. These markers can serve as candidate markers for future research, such as genetic diversity and relatedness analysis of different populations (Table S6).

### 2.5. Characteristics of Validated Microsatellite Loci

The polymorphic markers obtained were further assessed using two natural *G. yamadae* populations, including 50 individuals from Shaanxi province and 46 individuals from Beijing, China (Table S7). The 22 microsatellite markers had allele numbers ranging from 2 to 9 with an average value of 6 per locus. The polymorphism information content (PIC) values ranged from 0.099 to 0.782 with an average value of 0.48. The observed (H_O_) and expected (H_E_) heterozygosity ranged from 0.000 to 0.683 and 0.04 to 0.820, respectively. The inbreeding coefficient (F_IS_) ranged from −0.118 to 1.000. Hardy–Weinberg equilibrium (HWE) was tested in two geographical populations; 17 loci in the Shaanxi population and 18 loci in Beijing province departed from HWE (*p* < 0.05) (Table 5). Among these loci, GY14, GY18, GY19, GY27, GY42, GY66, GY67, GY68, GY70, GY72 and GY79 showed significant departures from HWE in both populations (*p* < 0.001).

**Figure 2:**
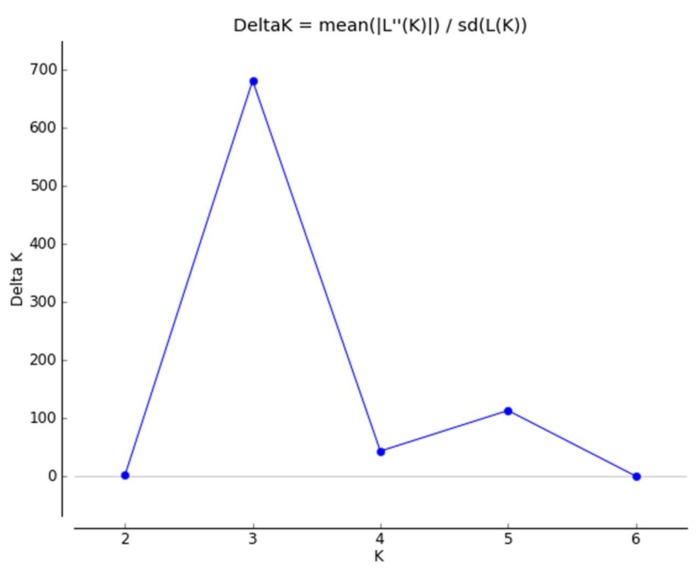
The model choice for each K value and graphical results of the SRUCTURE analysis.

**Figure 3:**
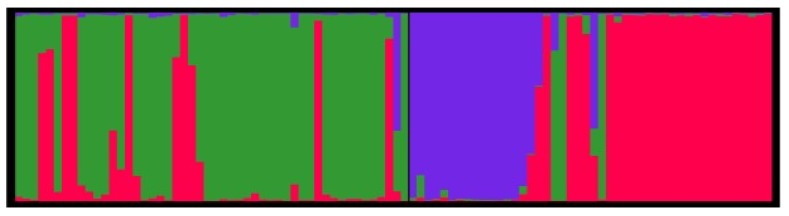
Population structure of K = 3 inferred by Bayesian clustering approaches based on 22 microsatellites markers.

The Bayesian algorithm implemented in the program STRUCTURE indicated that the model K = 3 explained the data in a satisfactory manner, because this model resulted in the highest ΔK value (Figure 2). The population structure of *G. yamadae* was inferred with the dataset of 22 microsatellite markers, and the 96 individuals from two geographical populations were grouped into three clusters, as shown in Figure 3.

**Figure 4:**
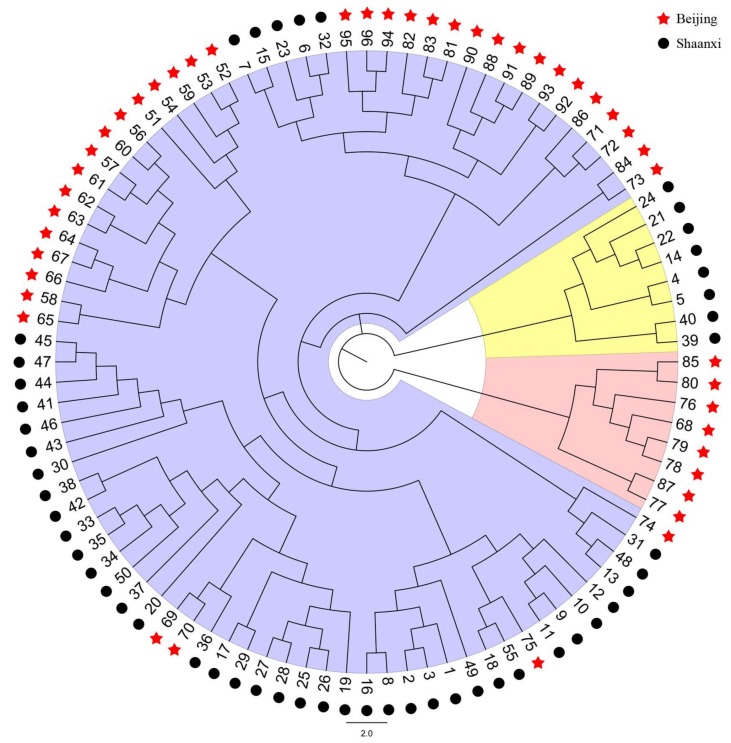
The unweighted pair-group method analysis (UPGMA) tree based on the genetic distance of two *G. yamadae* populations. Black circle represents individuals in Shaanxi province; red star represents Beijing individuals.

The topology of the unweighted pair-group method analysis (UPGMA) tree based on genetic distance shows the relationship between the two populations (Figure 4). Eight individuals from Shaanxi province and eight individuals from Beijing were grouped into independent clusters, which are highlighted in yellow and pink, respectively. These two clusters contributed 1/6 of the total tested individuals. The main cluster, marked in purple, is a mixed group that contains individuals from both geographical areas; however, sub-branches in this cluster were independent from each other and related to geographical distribution, with some exceptions, e.g., individuals 69, 70 and 75 from Beijing were clustered with the Shaanxi individuals (Figure 4).

## 3. Discussion

As one of the most ubiquitous pathogens of apples, *G. yamadae* has negative impacts on apple production as well as on cultivated junipers in China. However, the origin, introduction pathways and current population structure of *G. yamadae* remain unknown. The lack of a significant set of polymorphic microsatellite markers for *G. yamadae* and other *Gymnosporangium* species was one of the main justifications of the present study.

In this study, 22 polymorphic microsatellite markers were developed for *G. yamadae* based on transcriptome data sequenced by NGS. Microsatellites proved to be informative markers that can reveal heterozygotic or heterokaryotic genotypes in diploid or dikaryotic organisms [21,25,35]. Moreover, microsatellites are easy-to-use tools for population genetic and ecological studies as well as epidemiological and forensic research [27,29]. The characterization of SSRs requires the identification of genomic fragments containing repetitive sequence motifs [26]. Compared with the library-enriched method, recent advances in large-scale RNA-seq provide a fast, cost-effective and reliable approach for the generation of large expression datasets in non-model species [39,40,41]. We have described the efficient use of NGS to obtain a large amount of sequence data and applied bioinformatics tools to develop a novel sample of 3942 sequence-containing microsatellites for this species. The di-nucleotide types have generally been observed to have the highest frequency in many other rusts [30,31,32,33,34,35]. Compared with amplification in other fungi [22,27,28,29,30,31,32,33,34,35], the PCR amplification efficiency of the primers used here seems relatively low. *Gymnosporangium* species are demicyclic and we could not collect massive spores by inoculation experiments. Therefore, we extracted DNA directly from a sorus on infected host plants. Some specimens with few spores yielded relatively low-quality DNA and a low percentage of amplicons (48 out of 88).

The stabilization of genetic diversity is very important for the long-term interest of any species [42]. High level genetic diversity is related with a certain population size, for the loss of heterozygosity could have a negative effect on population fitness. However, the polymorphism information content (PIC) and alleles per locus were relatively low compared to those of other rusts [30,31,32,33,34,35], with average values of 0.48 and 6, respectively, which suggested that low genetic diversity was detected based on microsatellite markers. Furthermore, eleven microsatellite sites in both tested populations deviated significantly from Hardy–Weinberg equilibrium (*p* < 0.001), which may have resulted from heterozygote deficiency, because H_O_ is much lower than H_E_ in these loci (Table 5). Heterozygote deficiency can be caused by the presence of null alleles, small sample size, or the effects of population subdivision (Wahlund’s effect) [43].

The individuals from Beijing and Shaanxi were grouped into three main clusters. The largest cluster was a mixed group consisting of two geographical populations. One of the possible reasons for this observation is the frequent trans-provincial transportation of infected *Juniperus* seedlings all over the country. *J. chinensis* L. is one of the most popular landscaping plants in China and can be found in many areas, such as gardens, parks, roadsides, cemeteries and so on. However, during our collection in the field, we found that many seedlings with overwintering fungus galls had been recently planted locally, meaning that it is very likely that these seedlings were transported from another city or province. Usually, after a heavy spring rain, the telial horns extrude from the branch galls on the host juniper trees and become gelatinous; these structures can then produce basidiospores that are wind-blown to local *Malus* hosts. If the infection in *Malus* plants is successful, spermogonia develop in orange lesions on the upper surfaces of leaves. After a period of time, aecia also develop in the same orange lesions, but on the lower surfaces of the leaves. Mature aeciospores (spores produced in the aecia) are wind-blown to the juniper trees in the same year from early summer to fall. If infection is successful in the juniper host, a gall forms, and the telial horns grow from these symptomatic tissues the following spring or in subsequent years, concluding a complete life-cycle of *G. yamadae*. All this leads to a frequent regional gene exchange; thus, we could infer that some specimens collected in Beijing probably were from Shaanxi province or have an intimate relationship with those specimens.

Another likely reason for the observed clustering may be the limited number of specimens. The sample size should be large enough to make sure the results are an authentic reflection of the true ecological pattern rather than being an occasional phenomenon [44]. However, the sample size obtained in reality may be limited by many factors, such as budget, time and available samples. In a future study, we plan to collect many samples in each geographical area, and these samples would better cover enough regions where *G. yamadae* is potentially distributed to provide a more accurate conclusion about the genetic diversity of *G. yamadae* in China. Anyhow, microsatellites developed in this study are still effective molecular markers for detecting the polymorphism in *G. yamadae* populations.

Regardless of the above limitations, the microsatellites developed in this study will help researchers to better identify the main genetic groups in *G. yamadae* populations as well as the geographical relationships among geographically spaced populations. As a result, we will have novel insights into the importance of human activity in shaping the current structure of the *G. yamadae* population in China. Moreover, with the increasing genomic resources of *G. yamadae*, these informative SSR markers will help to identify virulence mechanisms associated with rust biology by mapping them onto the whole-genome sequences of populations segregated for virulence.

In summary, these highly informative SSR markers prove to be valuable molecular tools to study the aetiology of apple-juniper rust, which will help with the development of effective strategies for its management.

## 4. Materials and Methods

### 4.1. Rust Sample Collection

Samples of *G. yamadae* analysed in RNA-seq experiments were collected from an infected cypress tree (*Juniperus chinensis*.) prior to telial horn gelatinization at Northwest A & F University, Yangling, Shaanxi, China, in March 2016. Each sample was harvested in three biological replicates using liquid nitrogen and stored at −80 °C before RNA extraction. Each biological replicate was used for RNA extraction and RNA-Seq library construction.

The 96 samples prepared to be used to validate the polymorphism of *G. yamadae* microsatellites were collected in two geographic regions in China, of which 50 samples were from Shaanxi, and 46 samples came from Beijing. Likewise, all these samples were stored in absolute ethanol and frozen at −80 °C prior to use.

### 4.2. RNA Isolation and Library Preparation

An approximately 50 mg sample of teliospores from each replicate was used for total RNA extraction following a previously described protocol [45], and total mRNA was then purified using oligo (dT) magnetic beads. cDNA libraries were prepared according to Illumina sequencing sample preparation protocols. In total, 3 paired-end cDNA libraries were constructed, and sequenced with an Illumina HiSeq 2000 platform by Novogene Bioinformatics Technology Co., Ltd. (Beijing, China), and 100-bp paired-end reads were generated.

### 4.3. Reprocessing of Illumina Raw Data and de Novo Transcriptome Assembly

Using Trimmomatic software [46], clean data were obtained by removing reads containing adapter, reads containing ploy-A sequences (≥10%) and low quality reads (sQ ≤ 5) from raw data. Simultaneously, the Q20, Q30, GC content and sequence duplication level of the clean data were calculated. Transcriptome assembly was accomplished using Trinity software [37] with default parameters with min_kmer_cov set to 2 by default and all other parameters were set to the default values.

### 4.4. SSR Detection and Development of Primers

The microsatellite isolation from the genomic sequences was conducted by MISA software [47], and the primers for selected loci were designed with Primer 3 [38], based on the following core criteria: G/C content between 40% and 70%, annealing temperature between 57 °C and 61 °C, a product size ranging from 100 bp to 280 bp. The remaining parameters were at default settings. All candidate SSR primer pairs were synthesized by Ruibiotech Ltd. (Beijing, China).

### 4.5. DNA Extraction, Primer Testing and Polymorphism Detection

First, to validate the SSR markers, the genomic DNA of the 96 samples were extracted from single spores by proteinase K digestion followed by a standard phenol-chloroform method.

Second, we randomly selected 64 primers with the basic principle of having no mono-nucleotide motif repeats and a large repeat number and added a PC tail (Primer tail C) (5′ CAGGACCAGGCTACCGTG 3′) to the 5′ end of all candidate forward primers. Eight *G. yamadae* individuals were selected for the initial test. Amplification was performed in a final 10 μL reaction mixture containing 0.5 μL of template DNA, 5 μL of Master Mix (Promega, Madison, WT, USA), 0.25 μL of forward primer (modified by the PC tail) at a final concentration 0.25 μM, 0.25 μL (10 μM) of reverse primer at a final concentration 0.25 μM, and 4 μL of ddH_2_O. The amplification programme was as follows: 4 min at 94 °C; 35 cycles of 30 s at 94 °C, 30 s at 56 °C, and 45 s at 72 °C; and a final 10-min extension at 72 °C. The PCR products were visualized by agarose gel (3%) electrophoresis. This step was a preliminary validation of whether these primers could amplify a PCR fragment.

Third, the polymorphism of the primers validated in the previous step was further tested using a capillary sequencer. The PCR programme for amplification was the same as above. Amplification was performed in a final volume of 10 μL, containing 0.5 μL of template DNA, 5 μL of Master Mix (Promega, Madison, WT, USA), 0.08 μL of forward primer (modified by the PC tail) at a final concentration 0.08 μM, 0.16 μL(10 μM) of reverse primer at a final concentration 0.16 μM, 0.32 μL of fluorescently modified PC tail (FAM(blue), Hex(green), Rox(red)), including different colours at a final concentration of 0.32 μM, and 3.94 μL of ddH2O. The amplicons were visualized using a Gel Doc EZ system (Bio-rad, Berkeley, California, USA) and precise allele band sizing was conducted on the ABI 3830xl DNA analyser (Applied Biosystems, Froster, CA, USA) with the GeneScan 500LIZ size standard (Applied Biosystems, Froster, CA, USA).

Finally, 96 samples from the two regions were used to validate the effectiveness of the primers screened by the above steps. The amplification mixture, programme, and analysis of the PCR fragments were the same as in the second step.

### 4.6. Statistical Analysis

We used Popgene V1.32 [48] to calculate the number of alleles, observed heterozygosity (H_O_), expected heterozygosity (H_E_) and Shannon information index (I) and Powermarker V3.25 [49] to determine the inbreeding coefficient (Fis), polymorphism information content (PIC), deviations from the Hardy-Weinberg equilibrium (*HWE*), genetic distance and UPGMA tree based on the genetic distance. The possibility of the cryptic population structure of two geographical individuals were detected by STRUCTURE 2.2 [50]. Markov chain Monte Carlo (MCMC) consisted of a 100,000-iteration burn-in followed by 1,000,000 iterations. The simulated K values ranged from 2 to 7. Ten independent runs were implemented for each specific K value to verify the consistency of the results. The ad hoc estimated likelihood of K (ΔK) was used to determine the most likely number of populations (K) based on the rate of change in the log probability of the data (LnP(D)) [51].

## 5. Conclusions

In this study, we first characterized and developed several polymorphic microsatellite markers for *G. yamadae* from random regions of the transcriptome identified using next-generation sequencing. The new markers detected high levels of DNA polymorphism in *G. yamadae* and could be used to assess genetic diversity in apple-juniper rust. The loci assessed in our study revealed the genetic structure of two geographical populations. This approach provides a rapid method for high-throughput development of microsatellite markers from non-model species without reference genomes. These highly informative SSR markers will be potentially powerful tools to study genetic diversity and population structures of *G. yamadae* in China. In addition, they also facilitate a better understanding of the geographical origin and migration routes of *G. yamadae*, which will determine the influence human activity on the shaping of the population structures. Furthermore, comparative studies of markers mapped in populations of differentiating virulence can be used to identify virulence factors related to infection as well as develop apple resistance genotypes. Above all, these SSR markers will provide us with a relatively accurate understanding of the relationship within geographical populations and recent migration routes of *G. yamadae* in an upcoming study, which is crucial to understanding disease epidemics, developing risk-assessment models and slowing down the process of disease spread.

## Figures and Tables

**Table 1:** RNA-seq data statistics.

	DSXGY_1	DSXGY_2	DSXGY_3
**Raw reads**	47,629,874	59,428,164	49,485,212
**Clean reads**	46,917,624	58,748,388	48,752,948
**Q20 (%)**	97.18	96.52	97.34
**Q30 (%)**	93.82	91.47	93.42
**Mapped reads**	38,545,026 (82.29%)	50,102,470 (85.29%)	41,058,330 (84.35%)

DSXGY_1, DSXGY_2, DSXGY_3: three biological replicates of *G. yamadae.*

**Table 2:** Transcripts and Unigene length distribution statistics.

	Transcripts	Unigenes
200~300 bp	16,810	15,907
301~400 bp	5634	4855
401~500 bp	3124	2471
501~600 bp	2103	1471
601~700 bp	1692	1064
701~800 bp	1354	781
801~900 bp	1192	601
901~1000 bp	1100	551
1001~2000 bp	8601	3893
2001~3000 bp	4516	2069
3001~10 kbp	3180	1430
>10 kbp	13	9
total number	49,319	35,102
Max length	19,126	19,126
Average length	1006	756
N50	1957	1654
Total residues	49,601,912	26,521,164

**Table 3:** Statistics of microsatellites of different motif types and repeat numbers in *G. yamadae.*

Repeat Motif	Number of Repeat								Total Frequency (%)
	5	6	7	8	9	10	11	12	
A/T						1121	342	151	31.93%
C/G						131	92	47	5.34%
AC/GT		191	123	73	38	51	27	1	9.97%
AG/CT		198	92	64	43	33	38	3	9.32%
AT/AT		333	173	109	81	64	19	1	15.43%
CG/CG		8							0.16%
AAC/GTT	87	52	38	2					3.54%
AAG/CTT	86	31	16	3		1			2.71%
AAT/ATT	58	28	9	1		1			1.92%
ACC/GGT	136	46	30	2					4.23%
ACG/CGT	14	13	7	2				1	0.73%
ACT/AGT	37	15	21	3					1.50%
AGC/CTG	66	30	20	2					2.33%
AGG/CCT	67	16	14	2					1.96%
ATC/ATG	121	45	44	4					4.23%
CCG/CGG	26	3	7				1		0.73%
AAAC/GTTT	17	3							0.40%
AAAG/CTTT	4	2							0.12%
AAAT/ATTT	12	1							0.26%
AACC/GGTT	5	2							0.14%
AACG/CGTT		1							0.02%
AAGG/CCTT	1	1							0.04%
AAGT/ACTT									0.00%
AATC/ATTG	3								0.06%
AATG/ATTC	6	1							0.14%
ACAG/CTGT	4								0.08%
ACAT/ATGT	27	5							0.63%
ACCC/GGGT		2							0.04%
ACCG/CGGT	1								0.02%
ACCT/AGGT									0.00%
ACGC/CGTG	1								0.02%
ACTC/AGTG	1	1							0.04%
ACTG/AGTC	3	1	1						0.10%
AGAT/ATCT	2								0.04%
AGCC/CTGG	2	2	1						0.10%
AGCT/AGCT	1			1					0.04%
AGGC/CCTG	1	1			1				0.06%
AGGG/CCCT	3	1							0.08%
ATCC/ATGG	4	1	1						0.12%
OTHERS	32	13	10	8	5	1	2	1	1.42%
MNR						1252	434	198	37.27%
DNR		730	388	246	162	148	84	5	34.88%
TNR	698	279	206	21		2	1	1	23.90%
TTNR	98	25	3	1	1				2.53%
PNR	17	4	5		2				0.55%
HNR	15	9	5	8	3	1	2	1	0.87%

DNR: di-nucleotide repeats; TNR: tri-nucleotide repeats; TTNR: tetra-nucleotide repeats; PNR: penta-nucleotide repeats; HNR: hexa-nucleotide repeats.

**Table 4:** Frequency of different repeat motif in SSRs.

Number of Motif Copies	Mono-	Di-	Tri-	Tetra-	Penta-	Hexa-	Total	Frequency (%)
	0	0	0	0	0	0	0	0.00%
5	0	0	698	98	17	15	828	14.48%
6	0	730	279	25	4	9	1047	18.31%
7	0	388	206	3	5	5	607	10.62%
8	0	246	21	1	0	8	276	4.83%
9	0	162	0	1	2	3	168	2.94%
10	1252	148	2	0	0	1	1403	24.54%
11	434	84	1	0	0	2	521	9.11%
12	198	5	1	0	0	1	205	3.59%
13	119	0	0	0	0	0	119	2.08%
14	81	0	0	0	0	1	82	1.43%
15	68	0	0	0	0	1	69	1.21%
16	29	0	0	1	0	1	31	0.54%
17	41	0	1	1	0	0	43	0.75%
18	35	0	0	0	0	0	35	0.61%
19	56	0	0	0	1	0	57	1.00%
20	77	0	0	0	0	0	77	1.35%
21	74	0	0	0	0	1	75	1.31%
22	46	0	1	0	0	0	47	0.82%
23	23	0	0	0	0	0	23	0.40%
24	3	0	0	0	0	1	4	0.07%
Total	2536	1763	1210	130	29	49	5717	100.00%
Frequency (%)	44.36%	30.84%	21.16%	2.27%	0.51%	0.86%	100.00%	

**Table 5:** Characteristics of 22 microsatellite loci validated on 96 individuals of *G. yamadae* from two geographical locations in China.

Marker	Primer Sequences 5′-3′	Repeat Motif	Allele No.	Size Range (bp)	HWE		H_O_		H_E_		F_IS_		*PIC*	*I*
					Shaanxi	Beijing	Shaanxi	Beijing	Shaanxi	Beijing	Shaanxi	Beijing		
GY8	F: AAAAAGCATCAGGGGGAGGG	(CT)_6_	5	261–269	0.272	0.086	0.489	0.395	0.555	0.509	0.120	0.225	0.454	0.931
	R: GGTTGTGTGTGGCTAAGGCT													
GY14	F: ACAATCAACTAGAAATTGACCTTTGT	(AT)_6_	8	154–180	0.000	0.000	0.163	0.205	0.258	0.772	0.370	0.737	0.660	1.521
	R: TGGATGCAATATTTGAACTGTCAGA													
GY18	F: TCCAATCACCCCTCCCATCT	(TGT)_7_	7	219–237	0.001	0.000	0.250	0.048	0.391	0.203	0.363	0.767	0.290	0.688
	R: TCGAAAGTCGCAATACCAGCT													
GY19	F: TTCTTGAACGCAGACAGTGT	(GA)_9_	8	207–223	0.000	0.000	0.286	0.318	0.503	0.562	0.435	0.437	0.504	1.169
	R: TTCGCTCTCCCTCCCTCTTT													
GY20	F: ACGAGAGTGTCAGACGAAGC	(GCAAGA)_7_	6	195–225	0.213	0.294	0.633	0.683	0.702	0.698	0.100	0.022	0.650	1.358
	R: CTGACTTGTCGAGACCGAGG													
GY21	F: TCGTACAGCCACACACAGAC	(AC)_9_	2	120–122	0.752	0.018	0.511	0.293	0.470	0.470	−0.089	0.380	0.375	0.693
	R: AGAAGCACCATCGGTCAAGG													
GY25	F: GGCATGGAGAAAGCAGCAAG	(AG)_8_	3	141–145	0.000	0.017	0.300	0.278	0.496	0.482	0.432	0.427	0.376	0.718
	R: GCACCTCCAGGAAATCCCAA													
GY27	F: ACGTCCCTCAAATCTCATCCT	(TC)_9_	9	170–190	0.000	0.000	0.372	0.405	0.747	0.722	0.505	0.442	0.703	1.563
	R: CCGCCACGCTCAAAGAAAAT													
GY30	F: GATCAGGATGAGAGGCGGTG	(TC)_10_	4	214–230	0.017	1.000	0.102	0.231	0.118	0.207	0.137	−0.118	0.148	0.333
	R: TTGGTATGCATGCCAGGGAG													
GY42	F: TGTGGTTGTGGGGTTTTGGA	(AT)_9_	9	145–163	0.000	0.000	0.143	0.125	0.820	0.609	0.827	0.797	0.782	1.830
	R: ACCACACCACATCACATCATGT													
GY43	F: AGTGAAAGAGAGTGGATGTGC	(GT)_9_	4	154–162	0.010	0.036	0.061	0.065	0.099	0.105	0.385	0.384	0.099	0.256
	R: TGCGTCCCATGTATGTCTGT													
GY66	F: CCAGCATGCCTACCTAGCTG	(TGTGAT)_5_	4	181–199	0.000	0.000	0.000	0.000	0.547	0.649	1.000	1.000	0.598	1.198
	R: CAAGGAACAACAGCAGTGGC													
GY67	F: CGCGGTTCCGGATTGATAGA	(CGGAGT)_8_	4	190–208	0.000	0.000	0.000	0.000	0.682	0.611	1.000	1.000	0.612	1.218
	R: GGAATTCAGTCAAGGCCCCA													
GY68	F: TTCTTCTCTCCCAGCTCCCA	(CACTGC)_5_	7	129–177	0.000	0.000	0.333	0.075	0.620	0.643	0.465	0.885	0.621	1.345
	R: CAGTCAGCCTGTGTCCAGAG													
GY69	F: CTGGAGTCCGCCAATCAACT	(CCGCAT)_6_	5	203–227	0.779	0.122	0.396	0.366	0.355	0.523	−0.118	0.303	0.393	0.823
	R: ATTTTGGGACGAGCAGCTGA													
GY70	F: TGACCCCAATAAGACAAAAGTTGA	(ACAGCT)_9_	9	238–292	0.000	0.000	0.510	0.294	0.738	0.651	0.311	0.552	0.655	1.475
	R: GTTCAACTCCAGTCGGCTGA													
GY72	F: TACCACGAGTCCAGCTCTCA	(TGATCC)_5_	7	193–223	0.000	0.000	0.022	0.000	0.671	0.817	0.968	1.000	0.718	1.596
	R: AAAAGGAGTTGAGCGCGAGA													
GY75	F: CTCTGGCTCTGGCTTCAGTC	(CCTTAG)_6_	5	236–260	0.027	0.086	0.480	0.386	0.597	0.515	0.197	0.253	0.456	0.930
	R: AAGCCAAGCCAAGCAACTTG													
GY79	F: CGACAACTTTGCGCACTTGT	(TC)_10_	8	225–275	0.000	0.000	0.025	0.156	0.334	0.567	0.926	0.728	0.431	1.037
	R: ACTCCATTTTCTTGCATTTTGGGA													
GY81	F: ACCTCCCTGAAACACAAGCA	(CAC)_7_	5	245–263	0.004	0.000	0.265	0.378	0.372	0.449	0.289	0.159	0.352	0.740
	R: GCGTCTGAGTGGTGGAATCA													
GY86	F: TGTCGATTGGGATGGTTGGG	(GAAG)_5_	7	172–196	0.043	0.000	0.640	0.341	0.589	0.674	−0.087	0.497	0.568	1.250
	R: GTCCTCTCTGACTCGGGTCT													
GY87	F: ATCAGTGGCTCCCTCTCCAT	(CAT)_7_	6	233–263	1.000	0.001	0.041	0.073	0.040	0.207	−0.011	0.650	0.116	0.316
	R: GGGTAAGTGTTGGCGGAAGA													

F: forward primer; R: reverse primer; H_O_: observed heterozygosity; H_E_: expected heterozygosity; PIC: polymorphism information content; F_IS_: inbreeding coefficient; HWE: exact *p*-value of Hardy–Weinberg Equilibrium.

## Supplementary Materials

Supplementary materials can be found at http://www.mdpi.com/1422-0067/19/4/1178/s1.
