# Supplementary figures and images for: Development and Characterization of Novel Genic-SSR Markers in Apple-Juniper Rust Pathogen Gymnosporangium yamadae (Pucciniales: Pucciniaceae) Using Next-Generation Sequencing
Source: Int J Mol Sci. 2018 Apr 12;19(4):1178. doi: 10.3390/ijms19041178 (PMC5979324; doi:10.3390/ijms19041178)

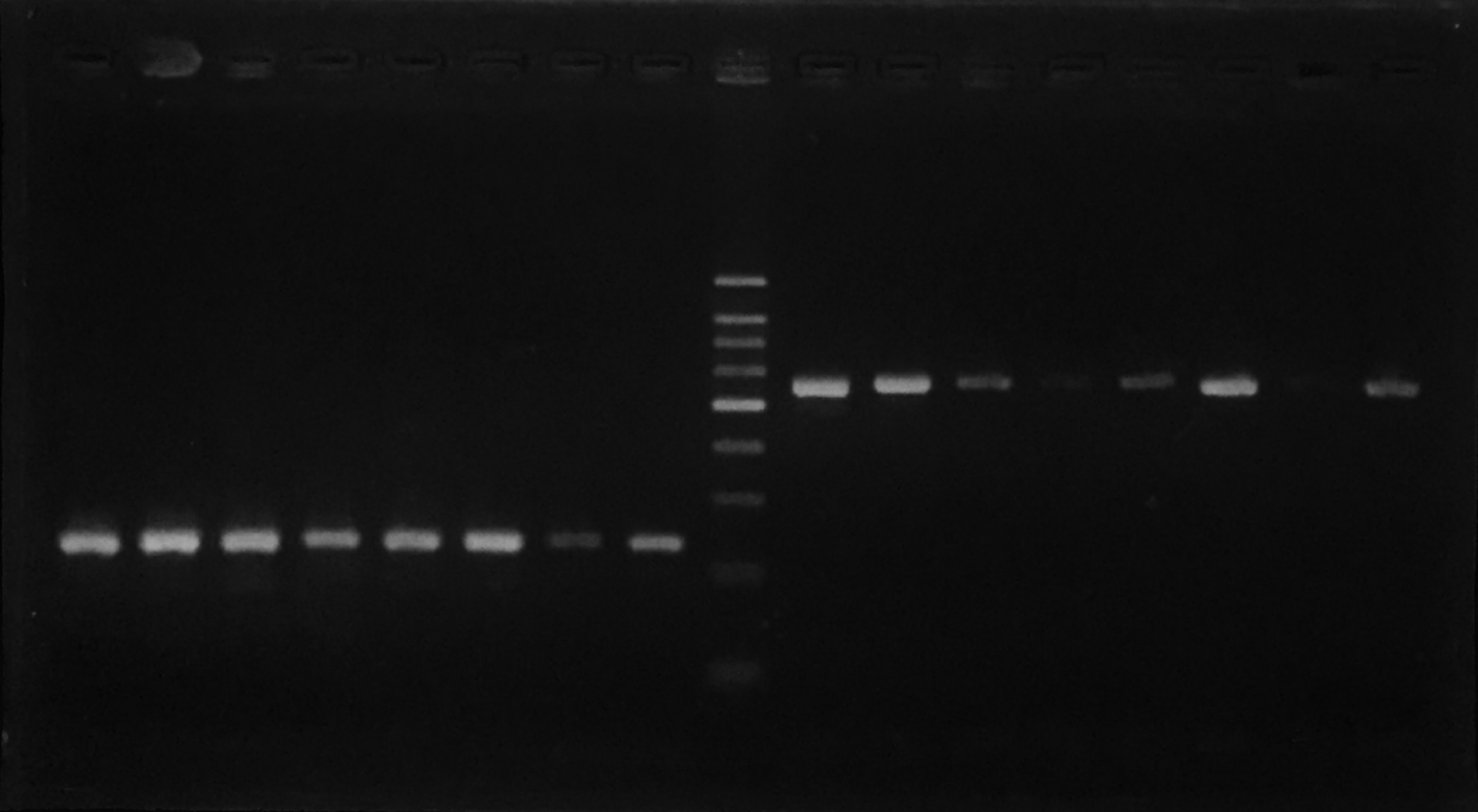

Supplement: Supplementary file 1 [file ijms-19-01178-s001.zip › ijms-284392-supplementary materials/GY1 and GY10.jpg]

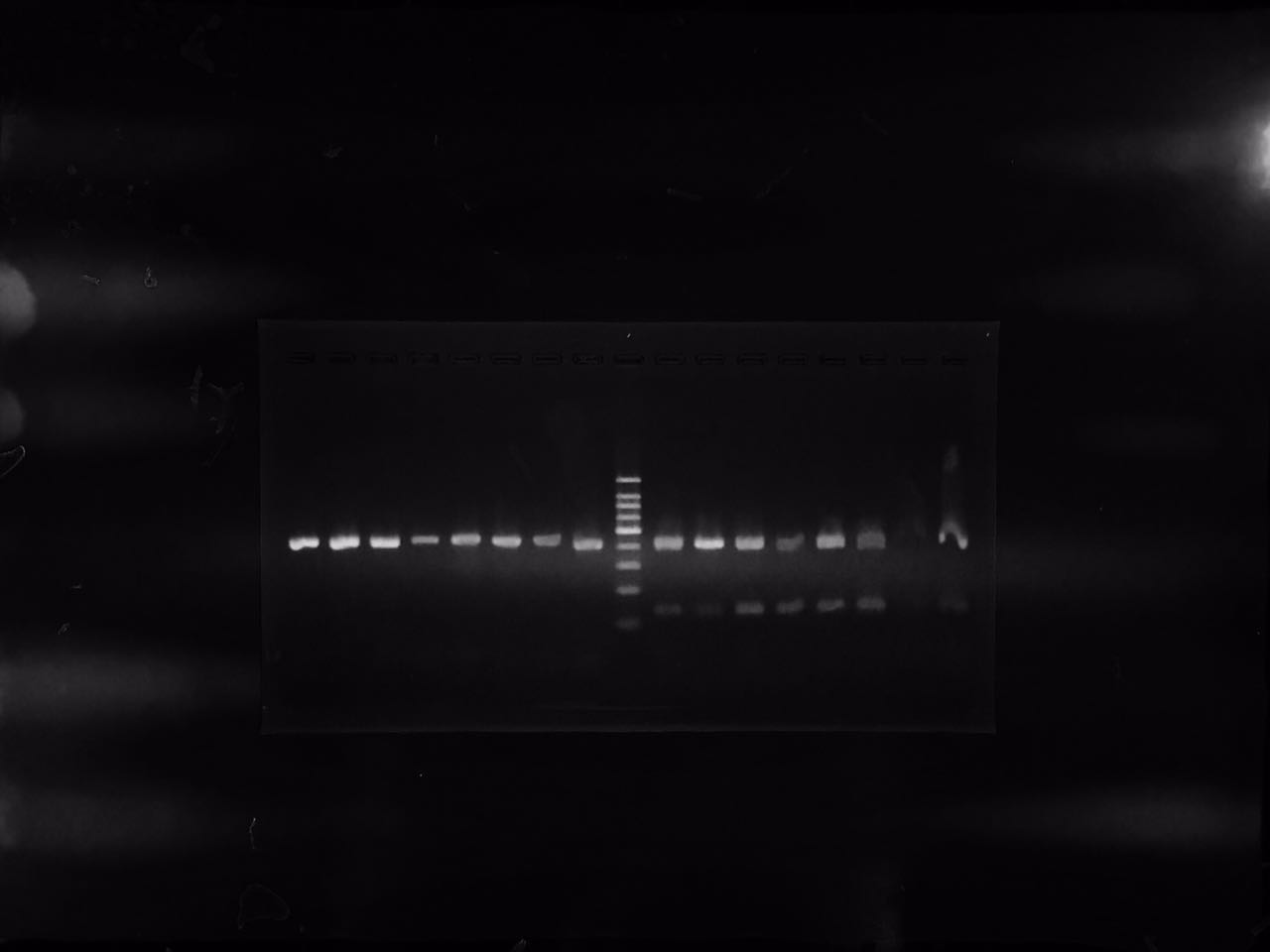

Supplement: Supplementary file 1 [file ijms-19-01178-s001.zip › ijms-284392-supplementary materials/GY13 and GY17.jpg]

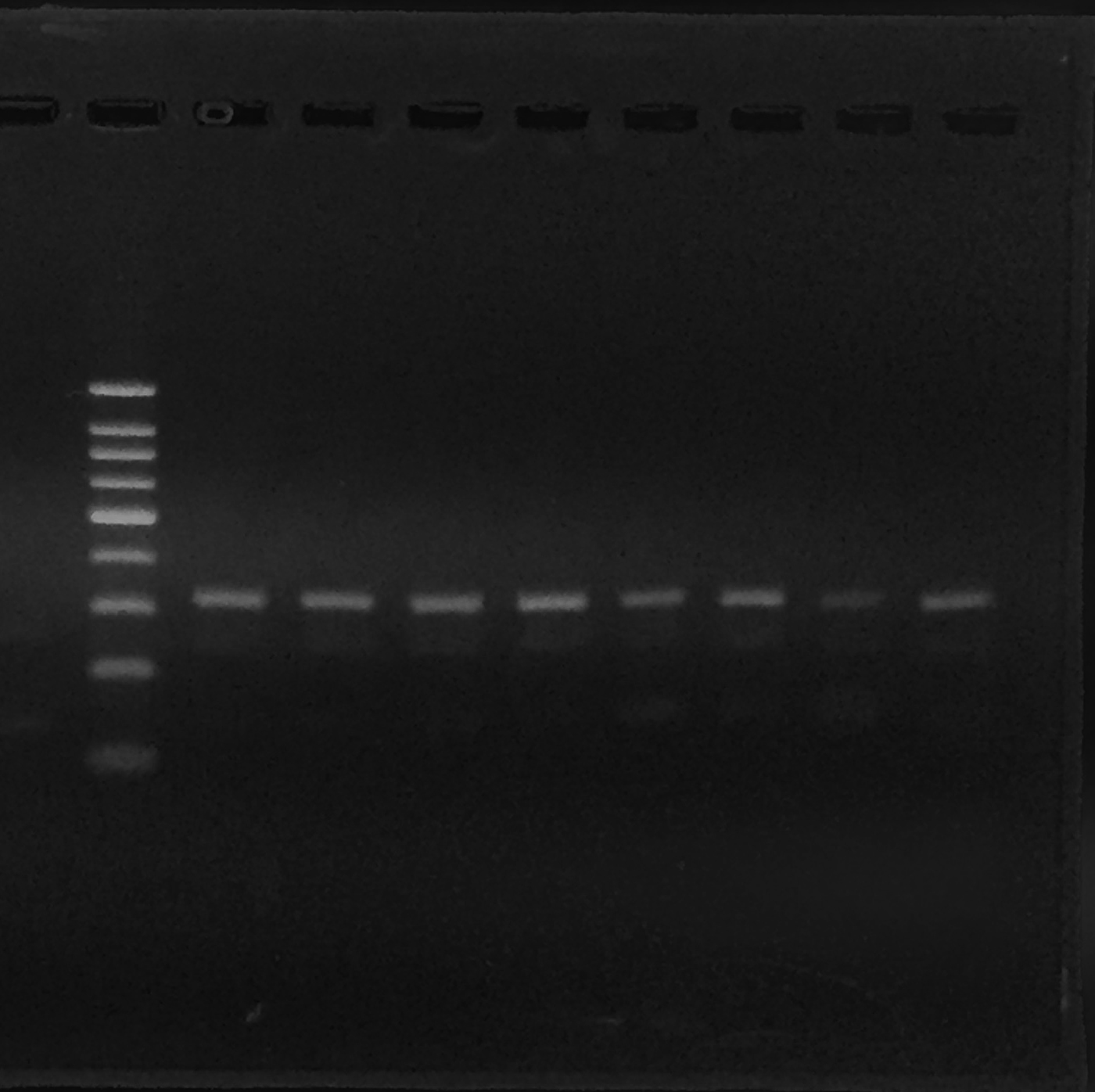

Supplement: Supplementary file 1 [file ijms-19-01178-s001.zip › ijms-284392-supplementary materials/GY14.jpg]

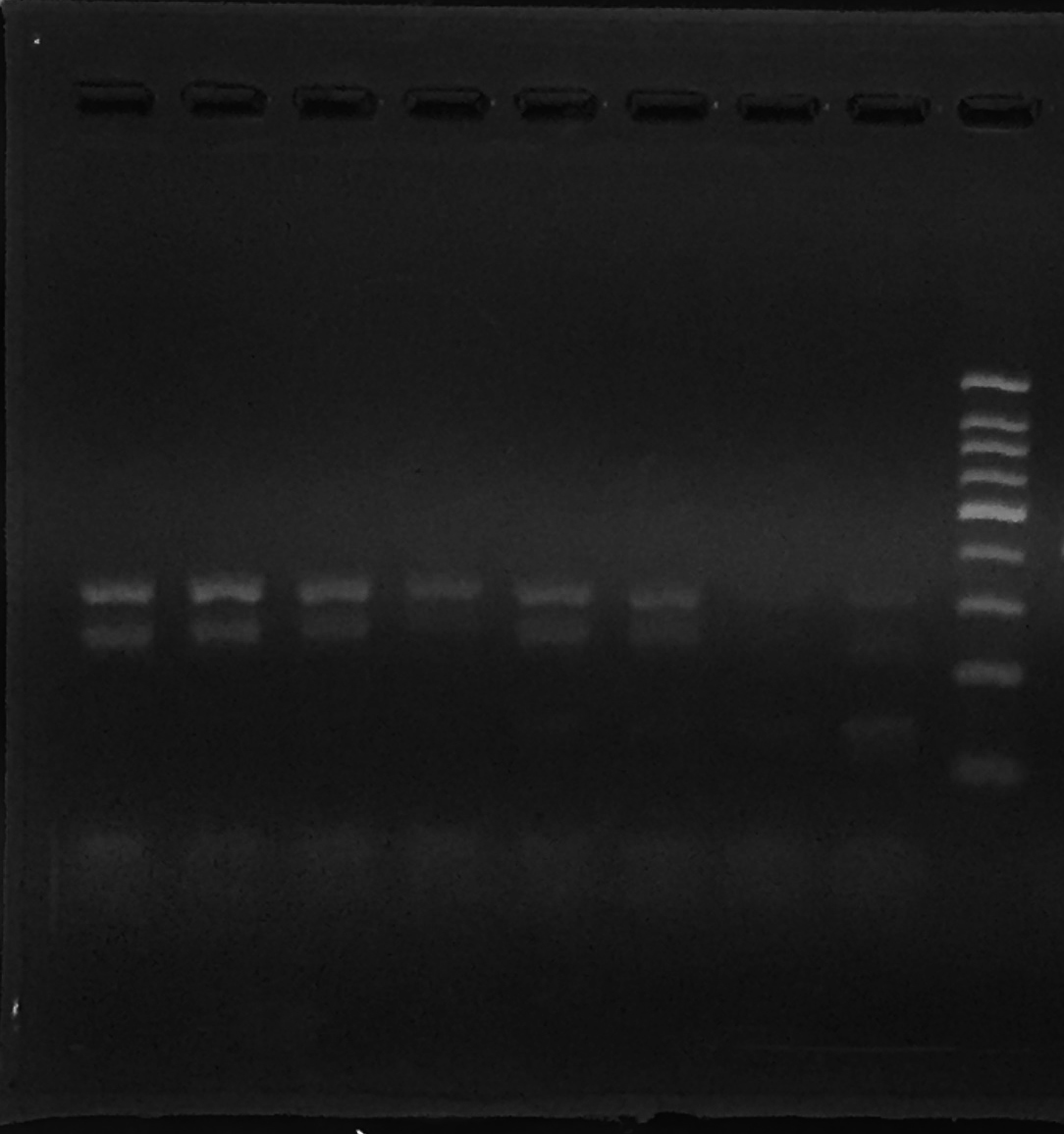

Supplement: Supplementary file 1 [file ijms-19-01178-s001.zip › ijms-284392-supplementary materials/GY15.jpg]

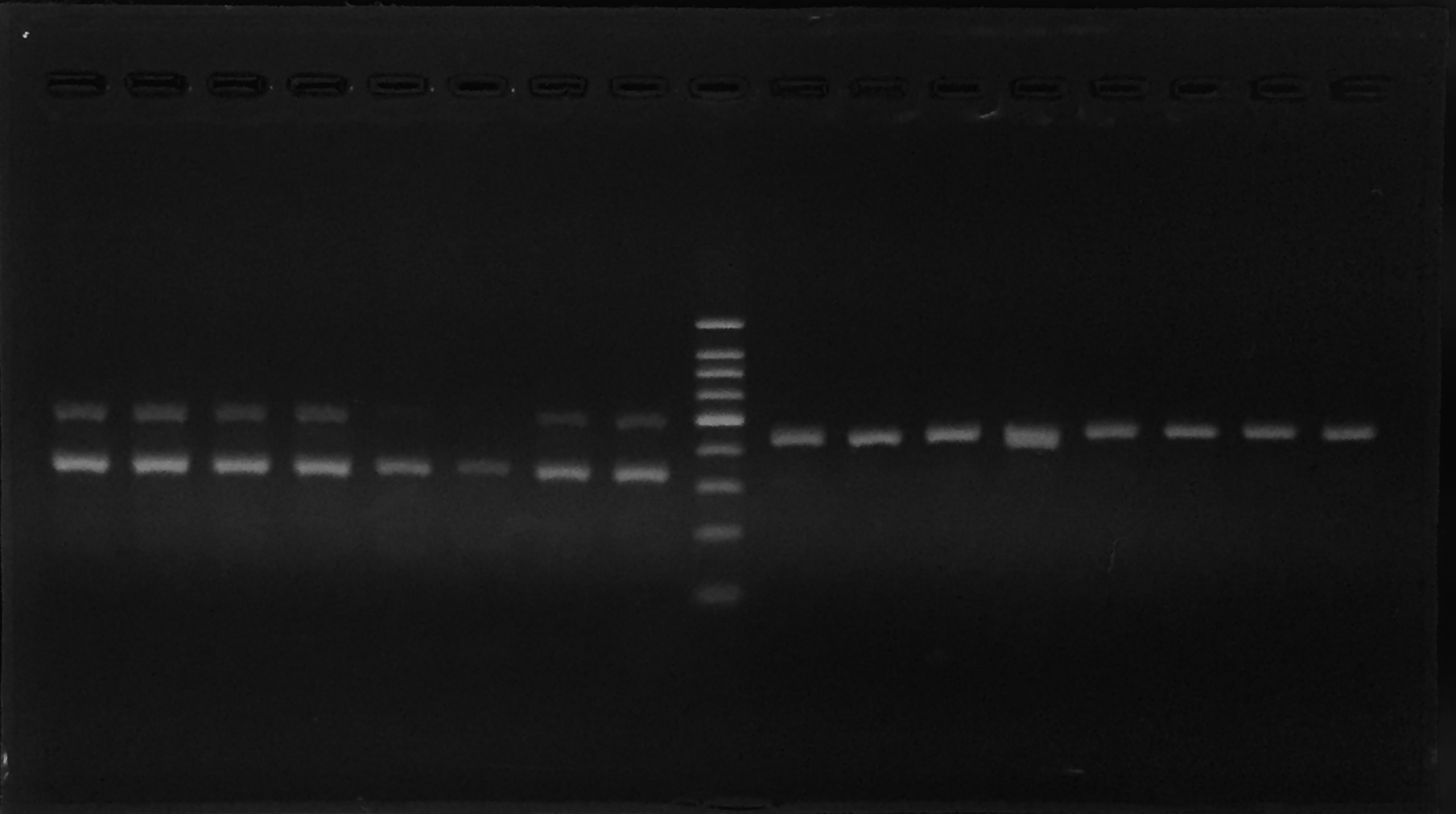

Supplement: Supplementary file 1 [file ijms-19-01178-s001.zip › ijms-284392-supplementary materials/GY16 and GY19.jpg]

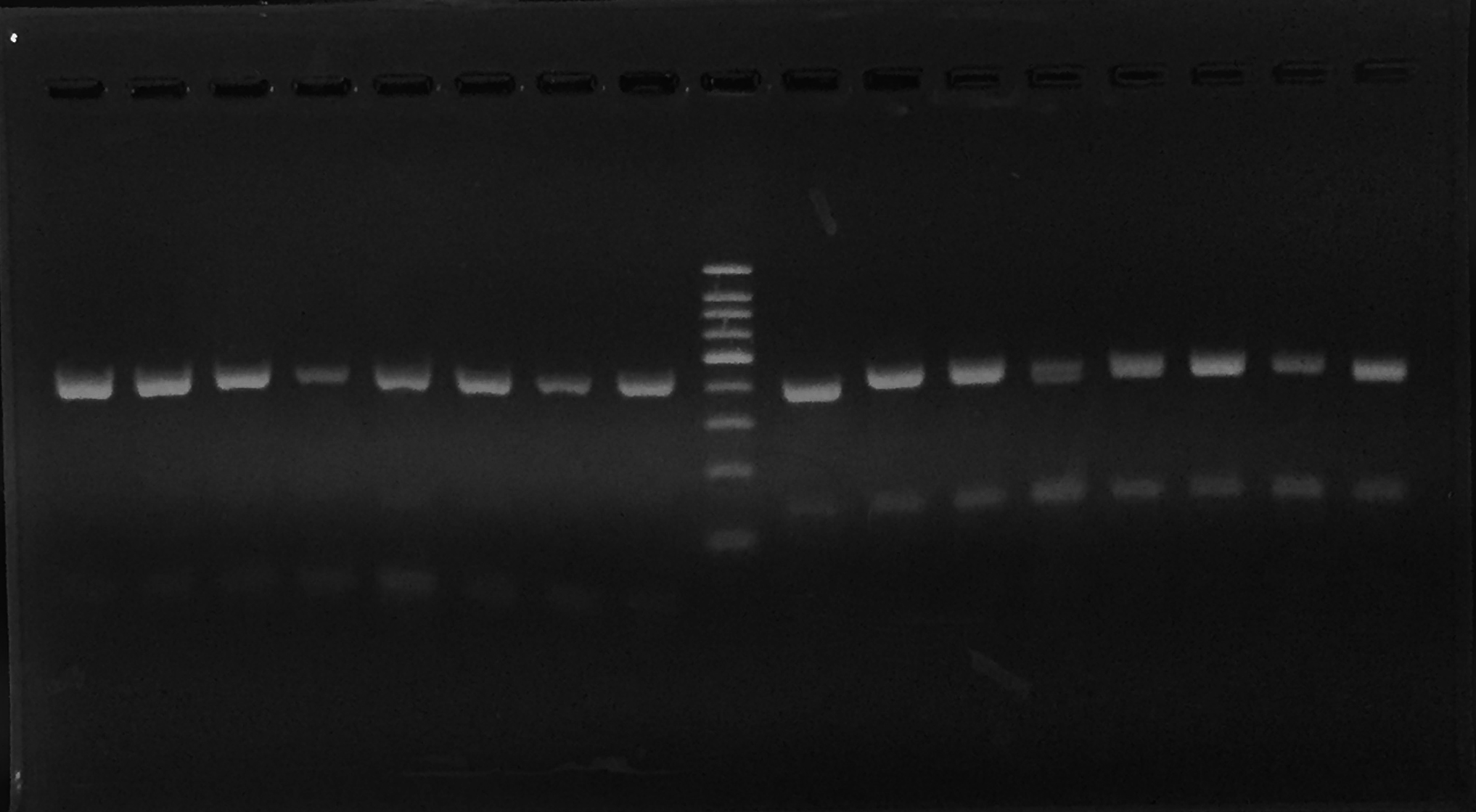

Supplement: Supplementary file 1 [file ijms-19-01178-s001.zip › ijms-284392-supplementary materials/GY18 and GY20.jpg]

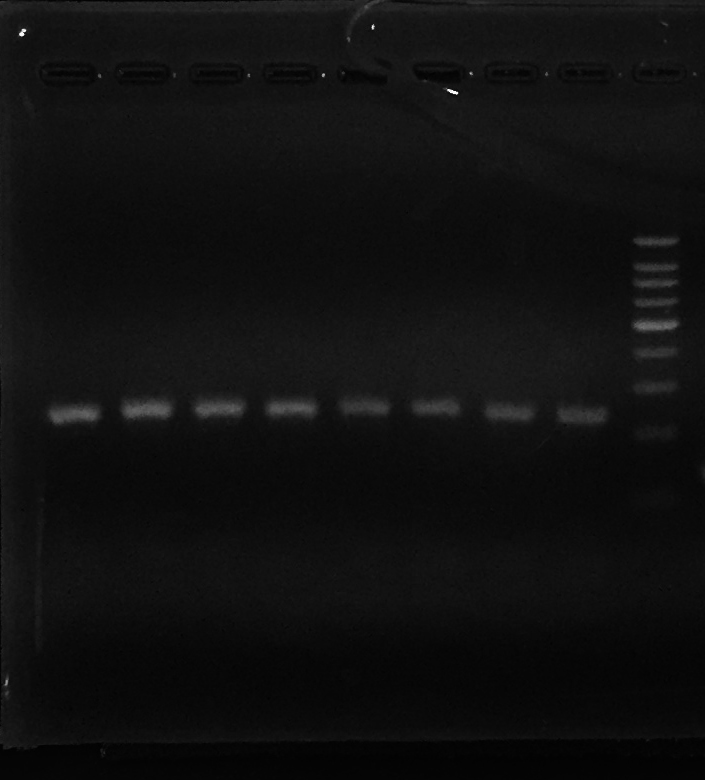

Supplement: Supplementary file 1 [file ijms-19-01178-s001.zip › ijms-284392-supplementary materials/GY21.jpg]

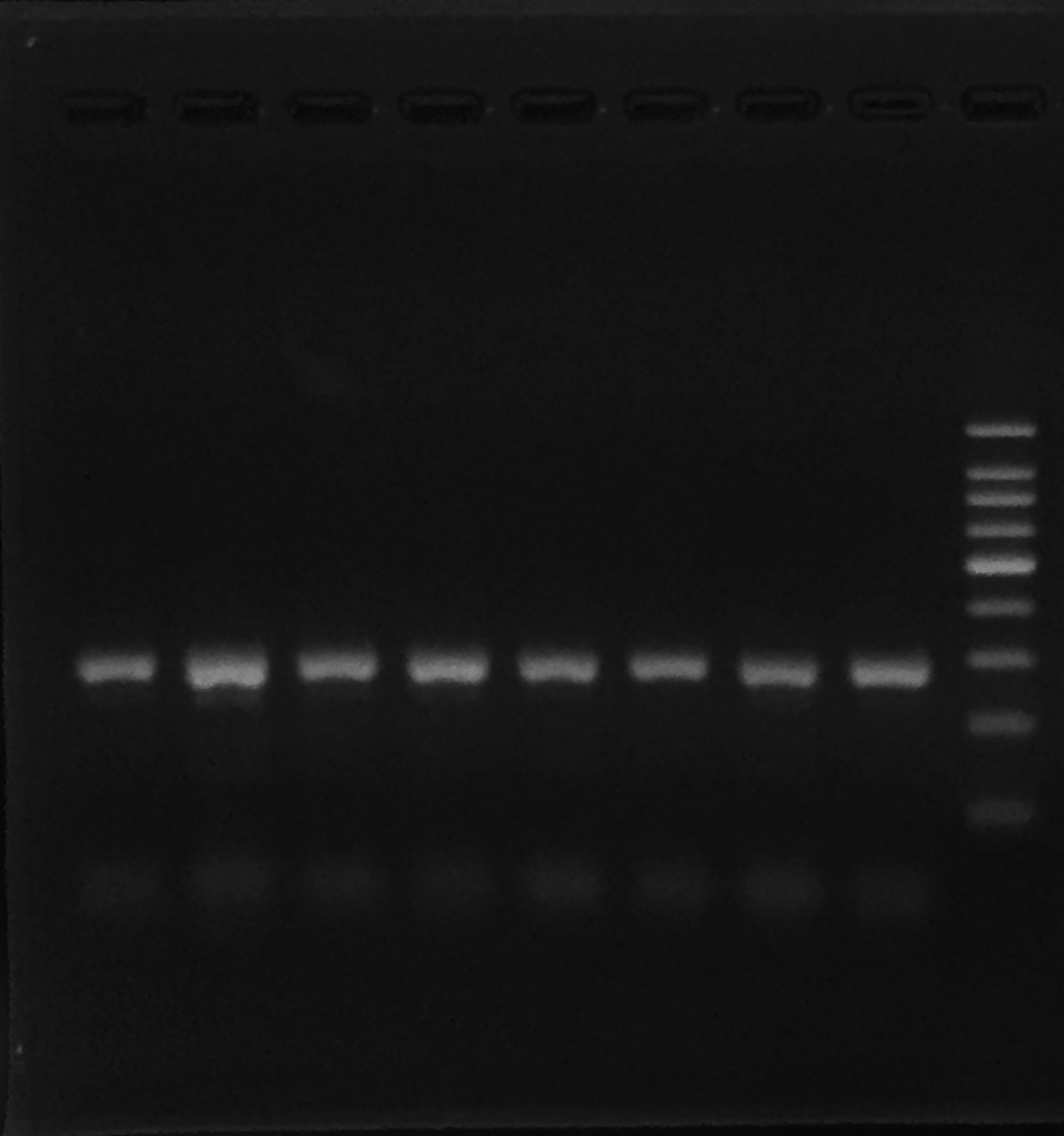

Supplement: Supplementary file 1 [file ijms-19-01178-s001.zip › ijms-284392-supplementary materials/GY25.jpg]

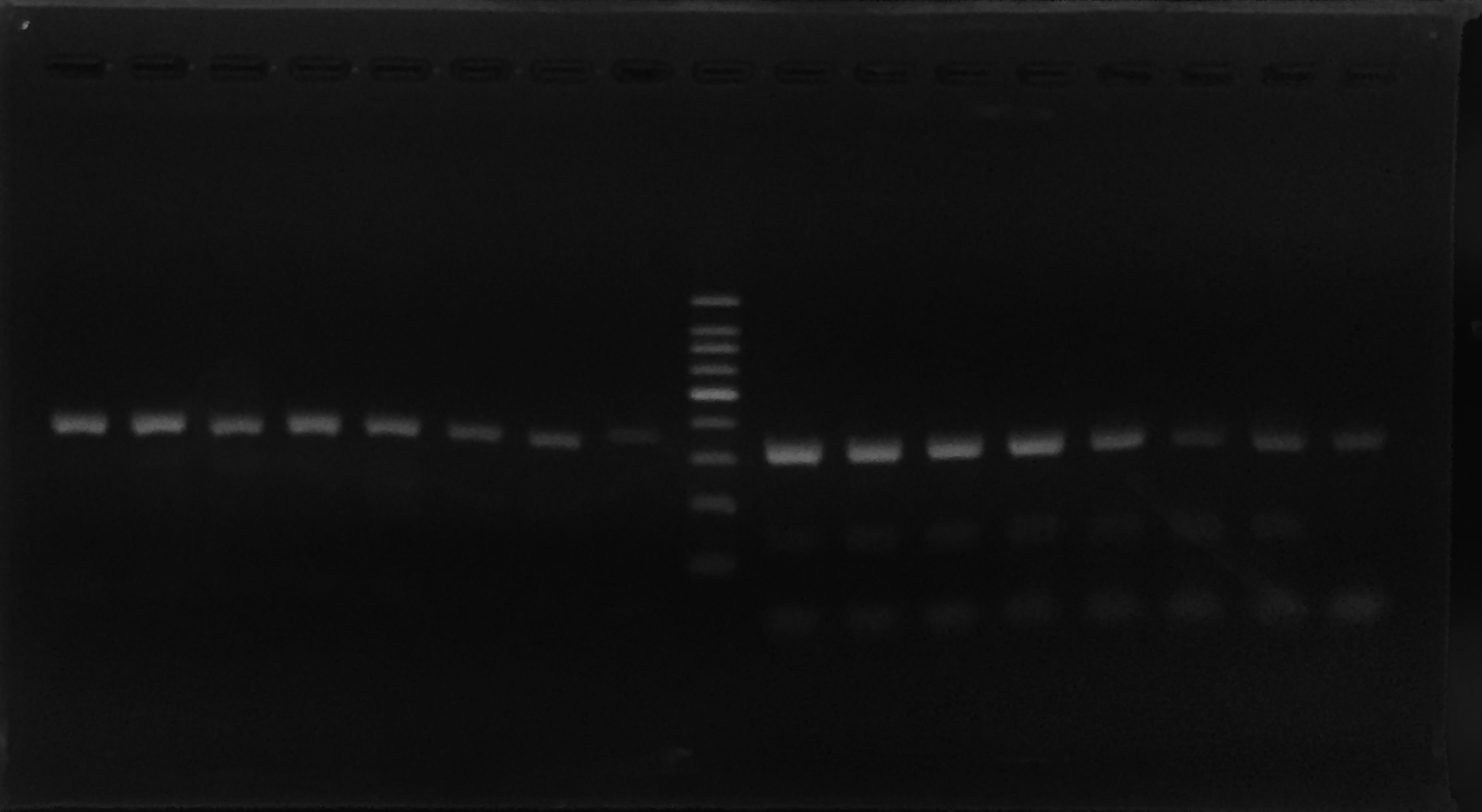

Supplement: Supplementary file 1 [file ijms-19-01178-s001.zip › ijms-284392-supplementary materials/GY27 and GY28.jpg]

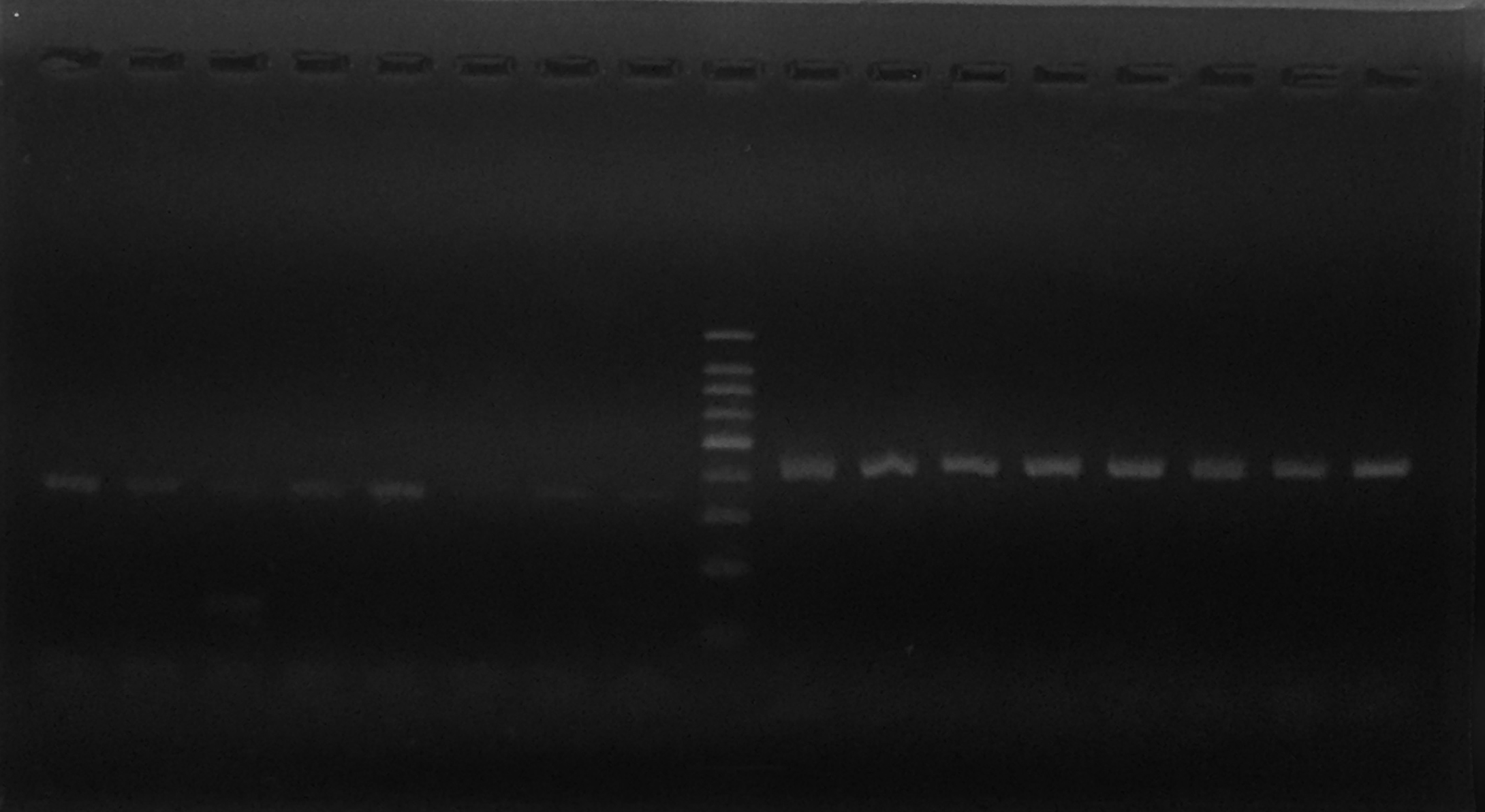

Supplement: Supplementary file 1 [file ijms-19-01178-s001.zip › ijms-284392-supplementary materials/GY29 and GY30.jpg]

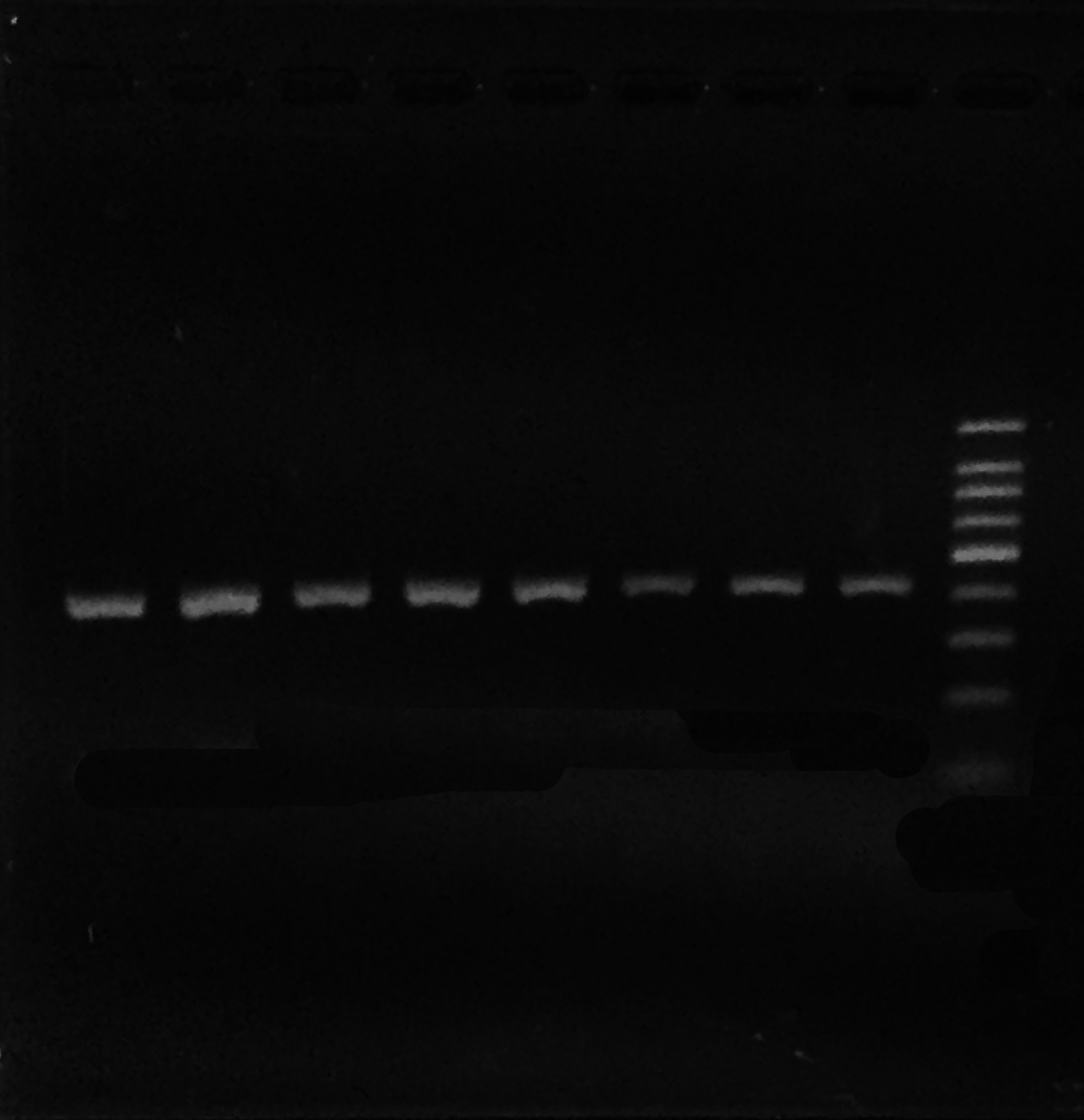

Supplement: Supplementary file 1 [file ijms-19-01178-s001.zip › ijms-284392-supplementary materials/GY31.jpg]

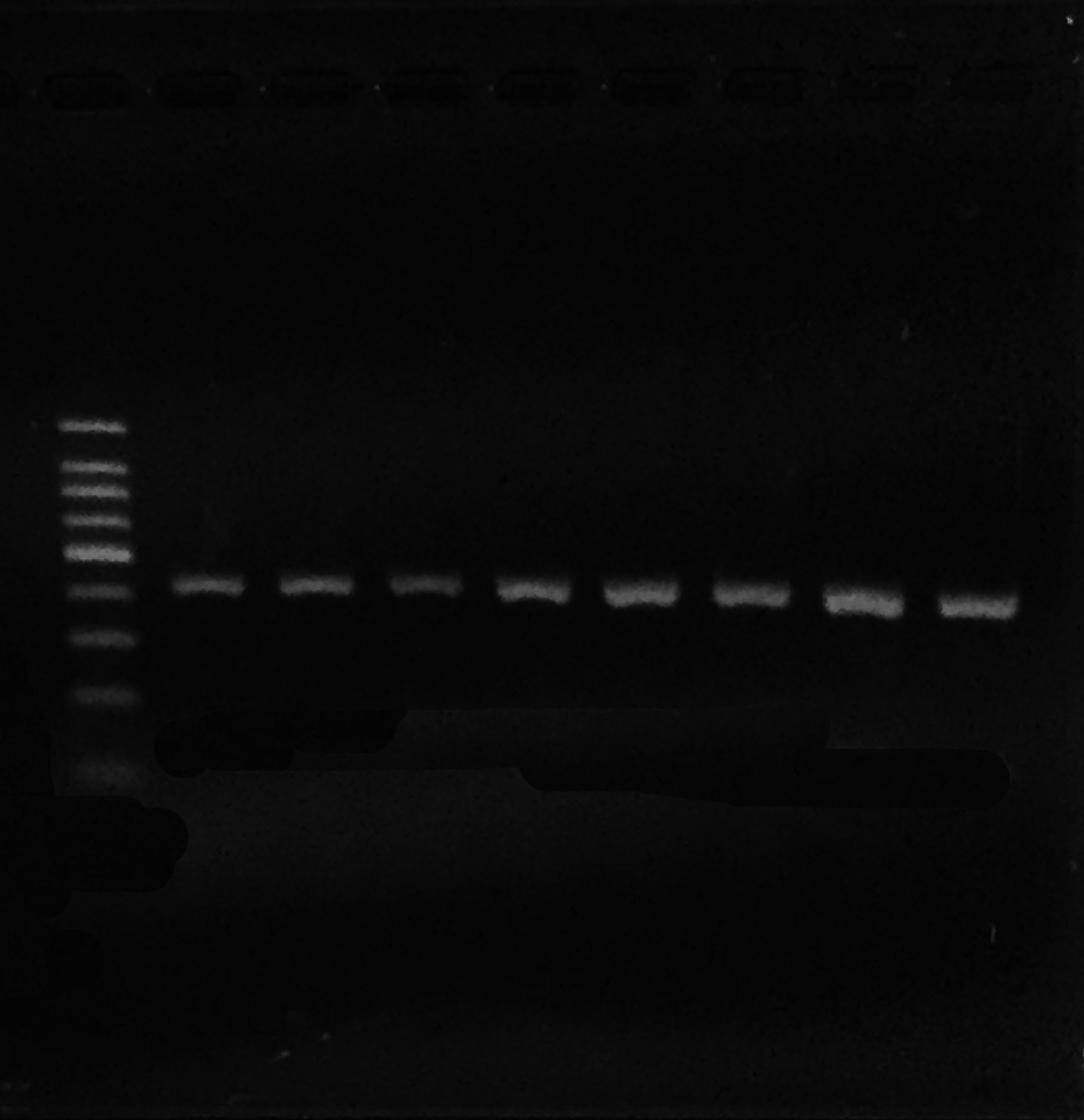

Supplement: Supplementary file 1 [file ijms-19-01178-s001.zip › ijms-284392-supplementary materials/GY32.jpg]

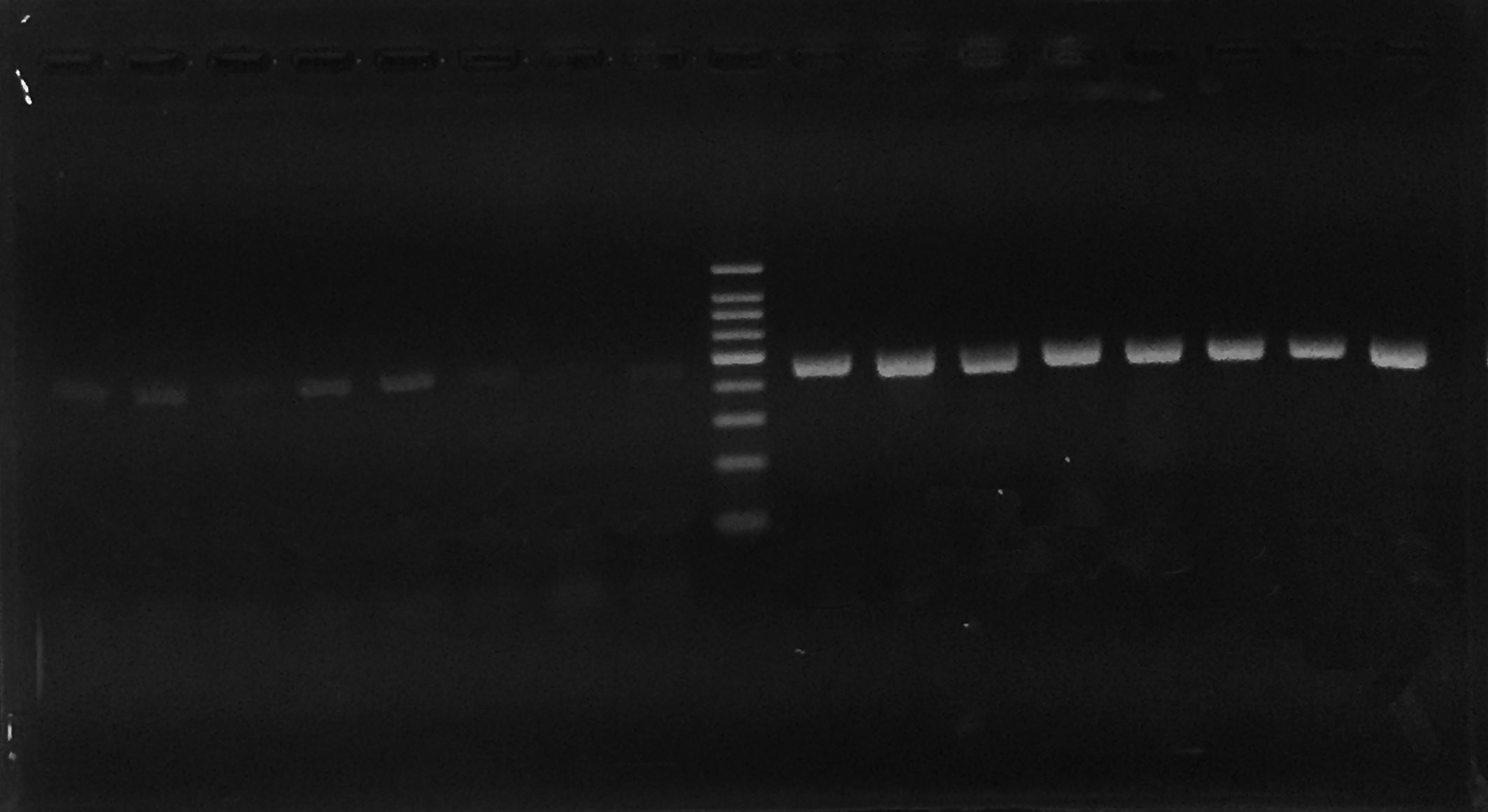

Supplement: Supplementary file 1 [file ijms-19-01178-s001.zip › ijms-284392-supplementary materials/GY35 and GY36.jpg]

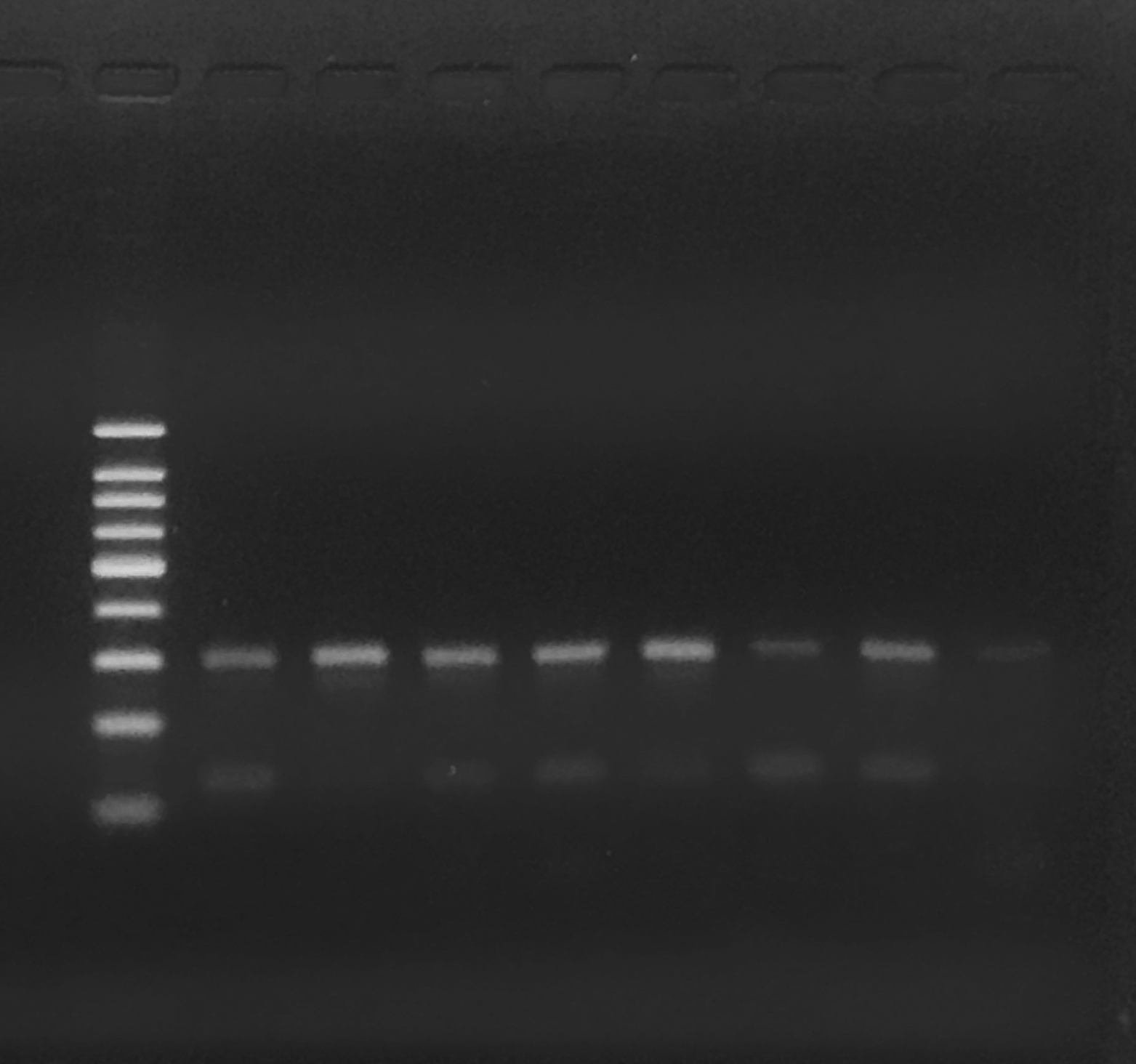

Supplement: Supplementary file 1 [file ijms-19-01178-s001.zip › ijms-284392-supplementary materials/GY42.jpg]

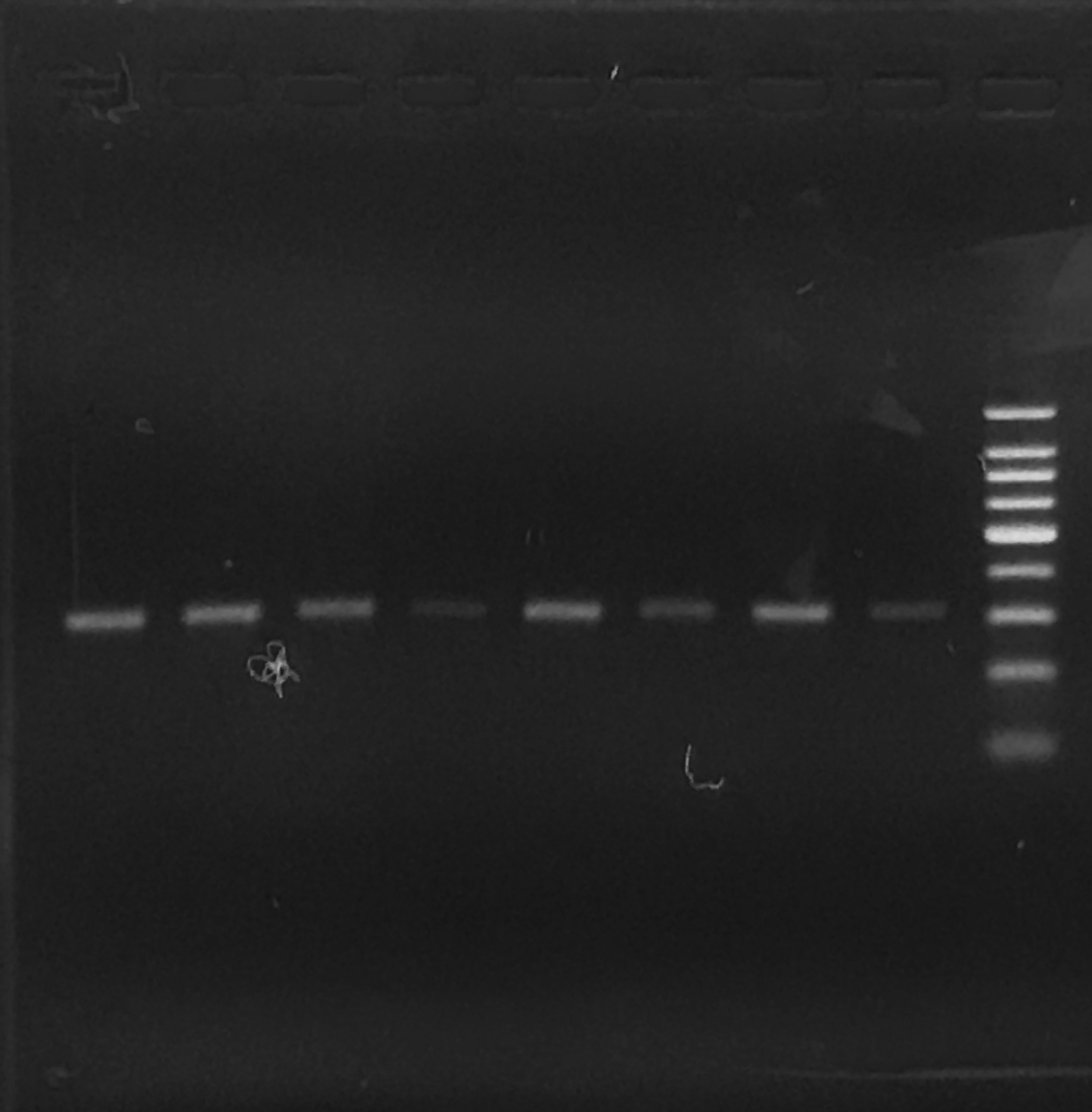

Supplement: Supplementary file 1 [file ijms-19-01178-s001.zip › ijms-284392-supplementary materials/GY43.jpg]

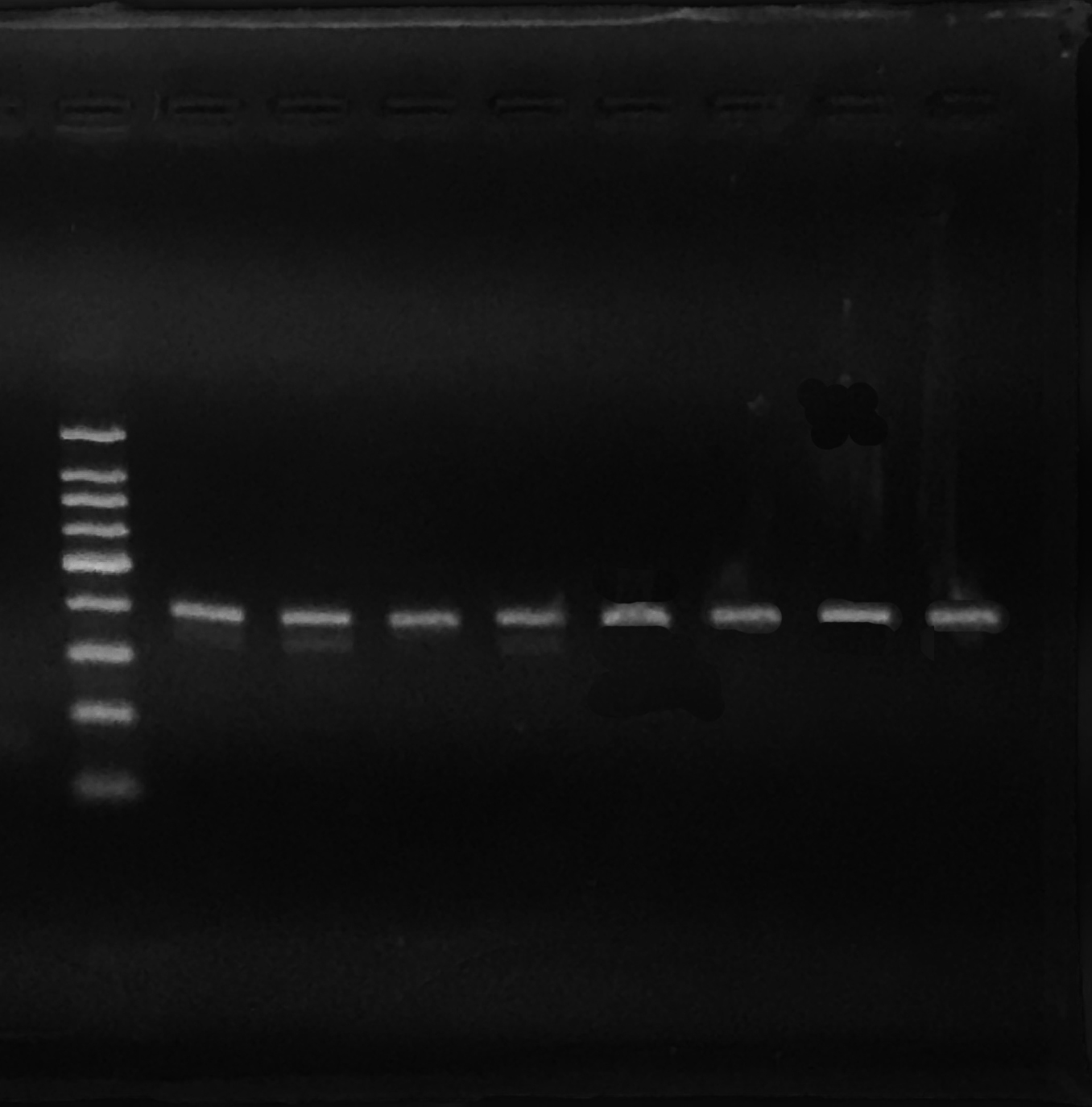

Supplement: Supplementary file 1 [file ijms-19-01178-s001.zip › ijms-284392-supplementary materials/GY46.jpg]

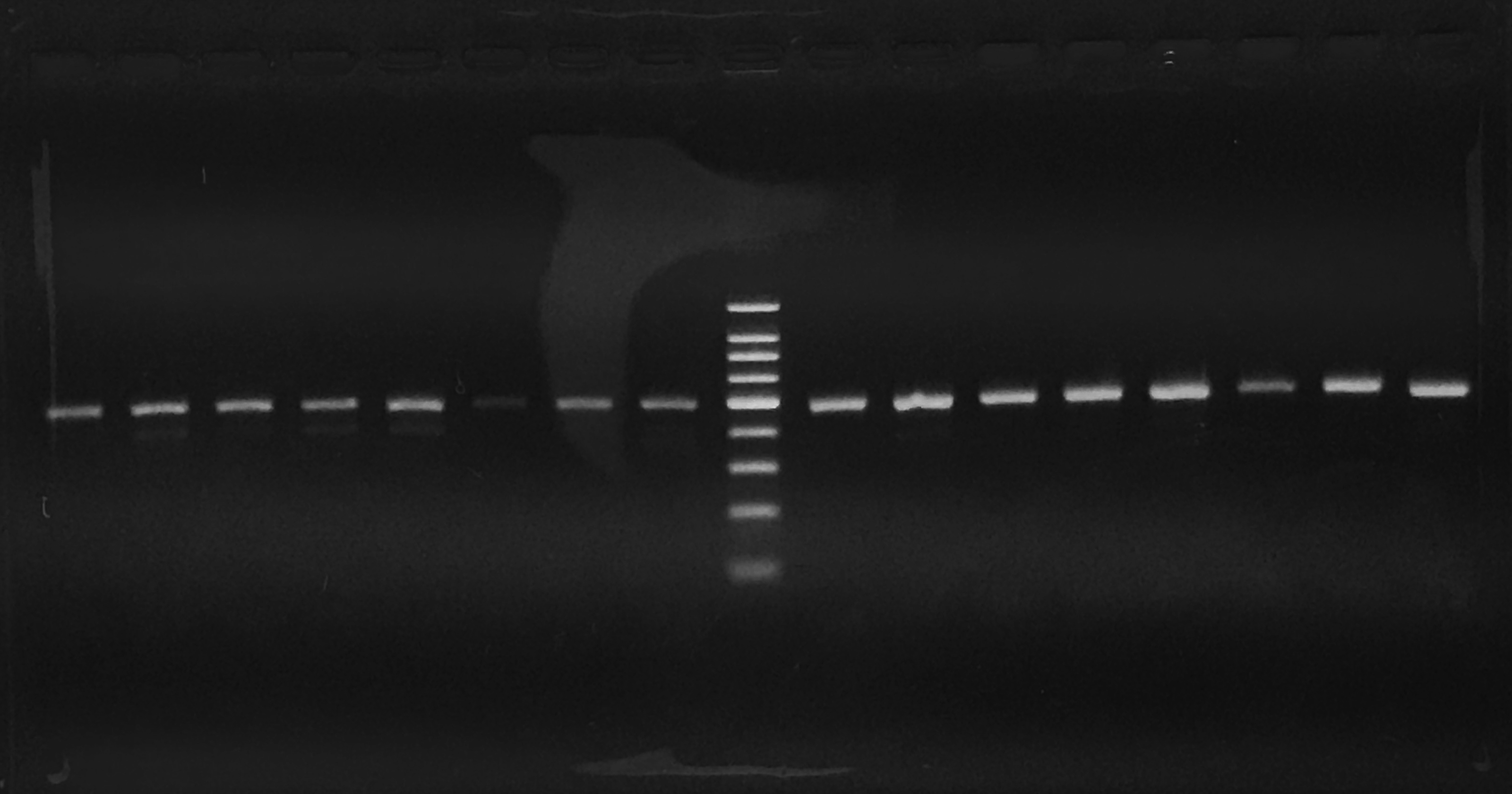

Supplement: Supplementary file 1 [file ijms-19-01178-s001.zip › ijms-284392-supplementary materials/GY47 and GY48.jpg]

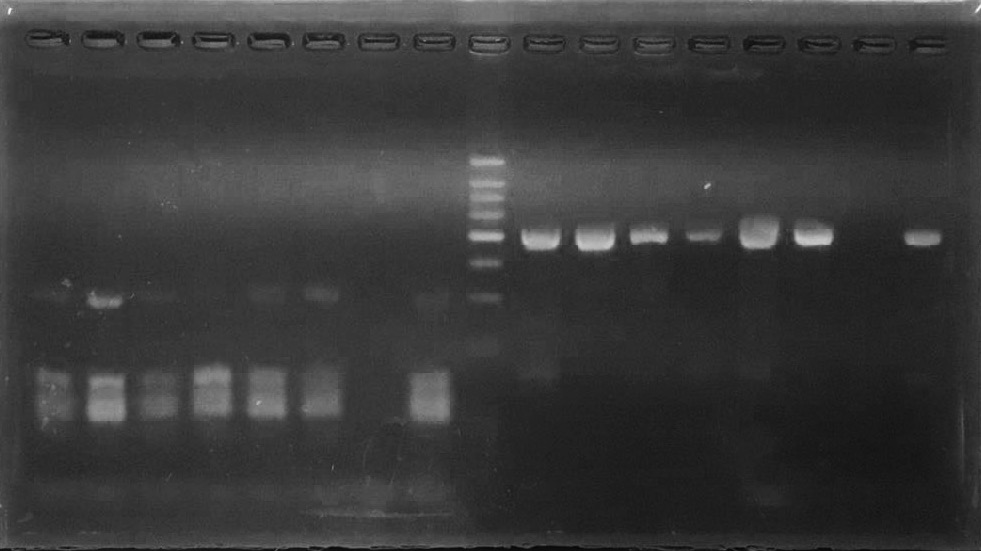

Supplement: Supplementary file 1 [file ijms-19-01178-s001.zip › ijms-284392-supplementary materials/GY5 and GY8.jpg]

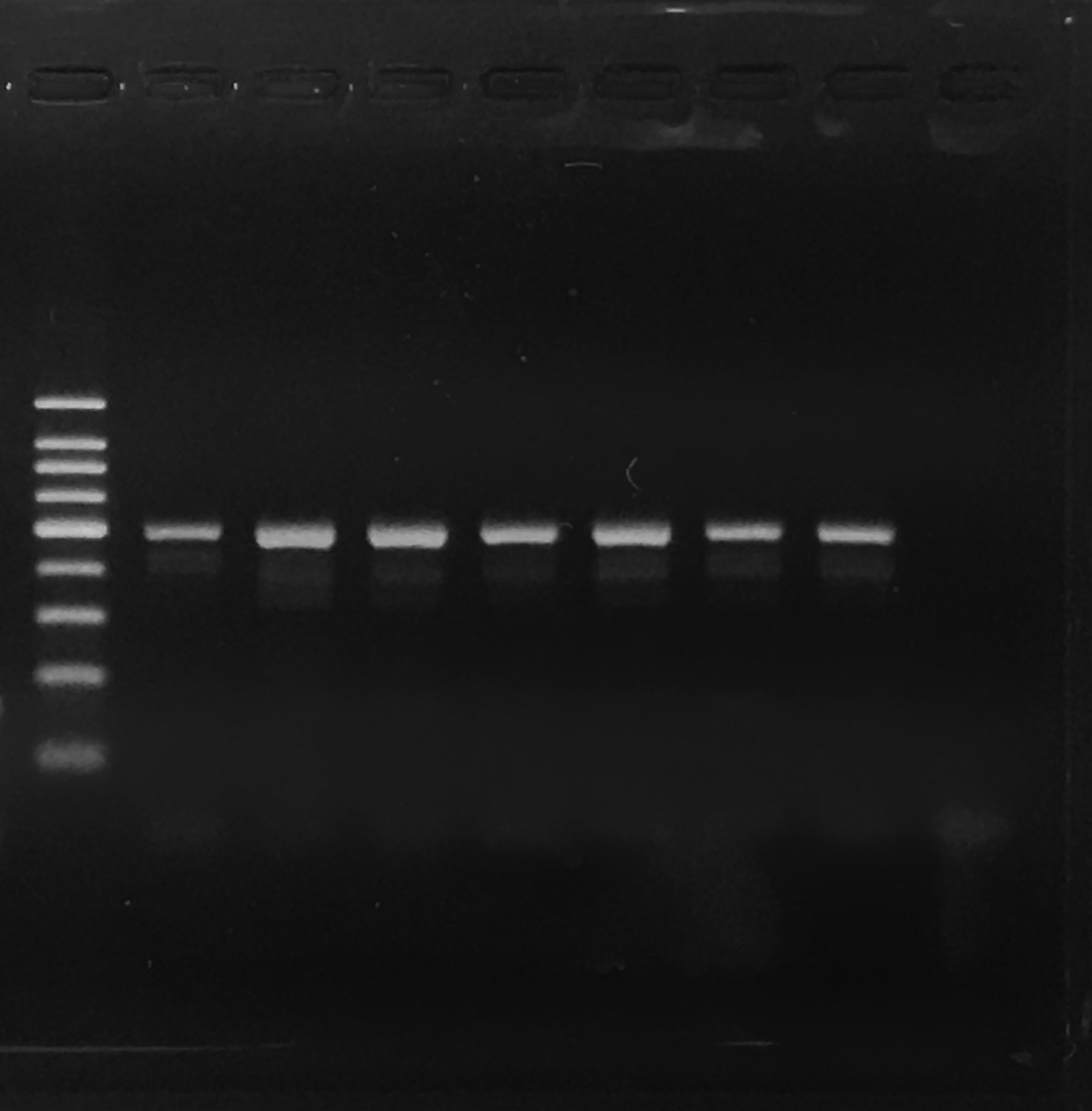

Supplement: Supplementary file 1 [file ijms-19-01178-s001.zip › ijms-284392-supplementary materials/GY50.jpg]

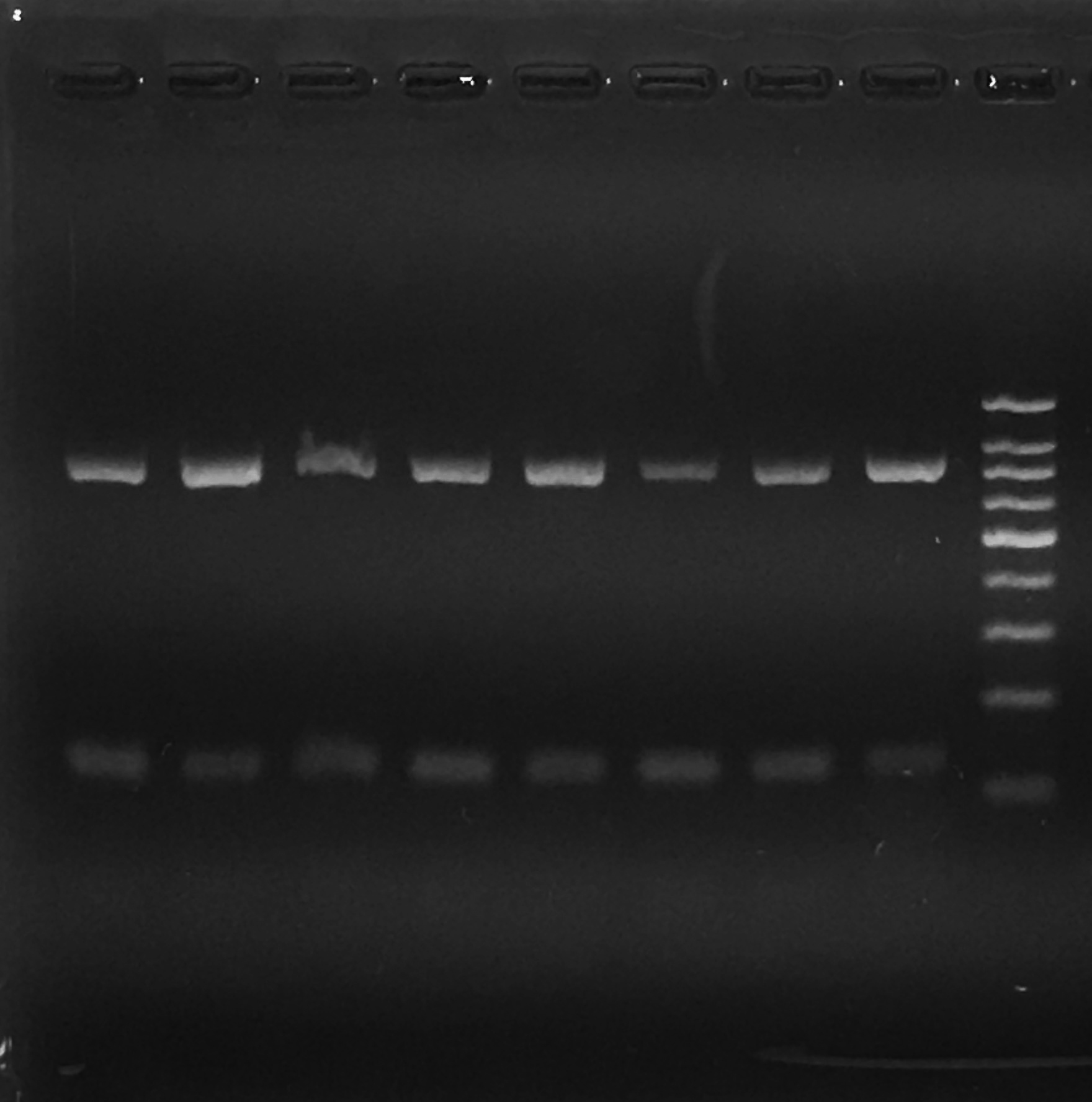

Supplement: Supplementary file 1 [file ijms-19-01178-s001.zip › ijms-284392-supplementary materials/GY51.jpg]

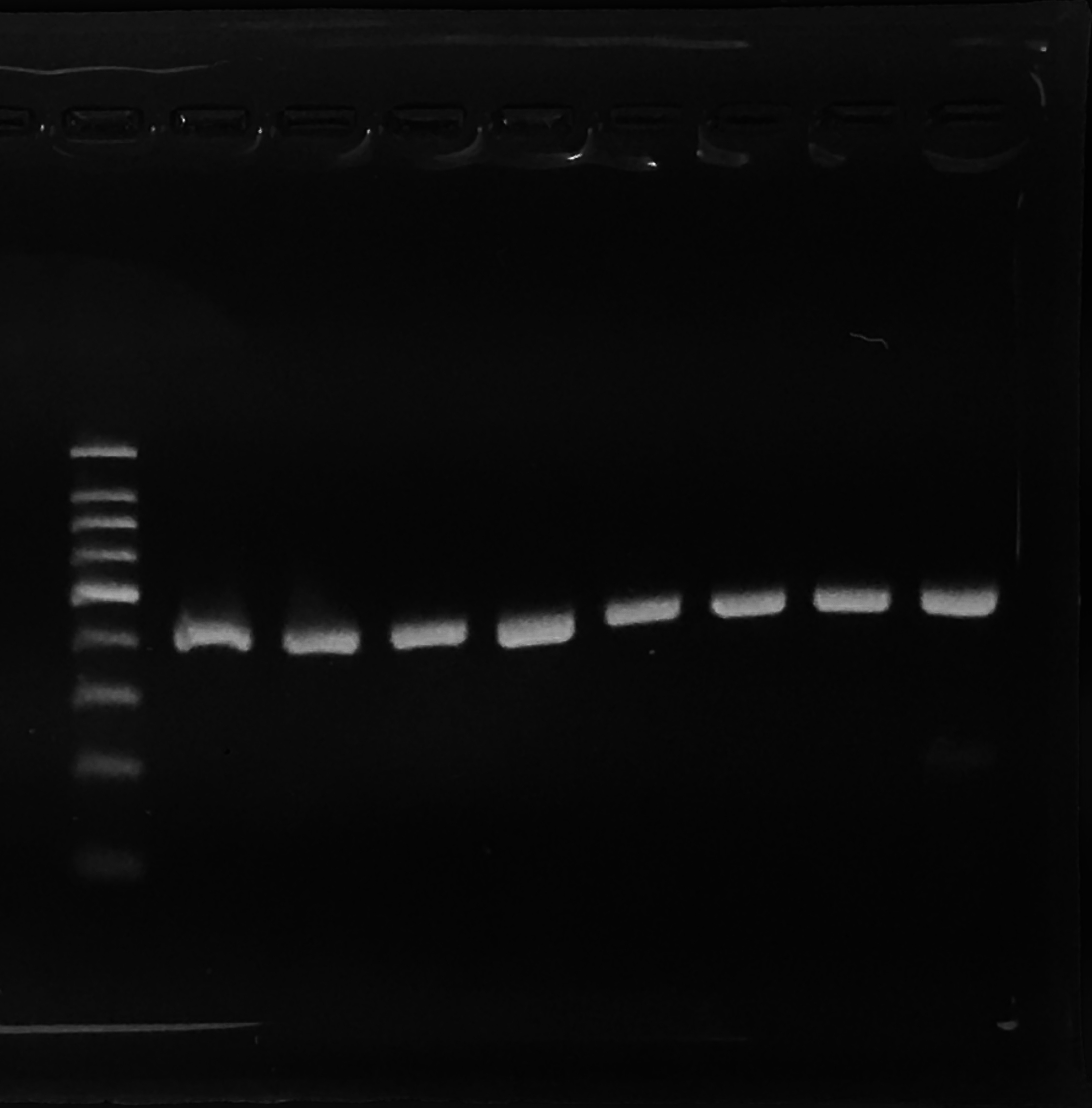

Supplement: Supplementary file 1 [file ijms-19-01178-s001.zip › ijms-284392-supplementary materials/GY58.jpg]

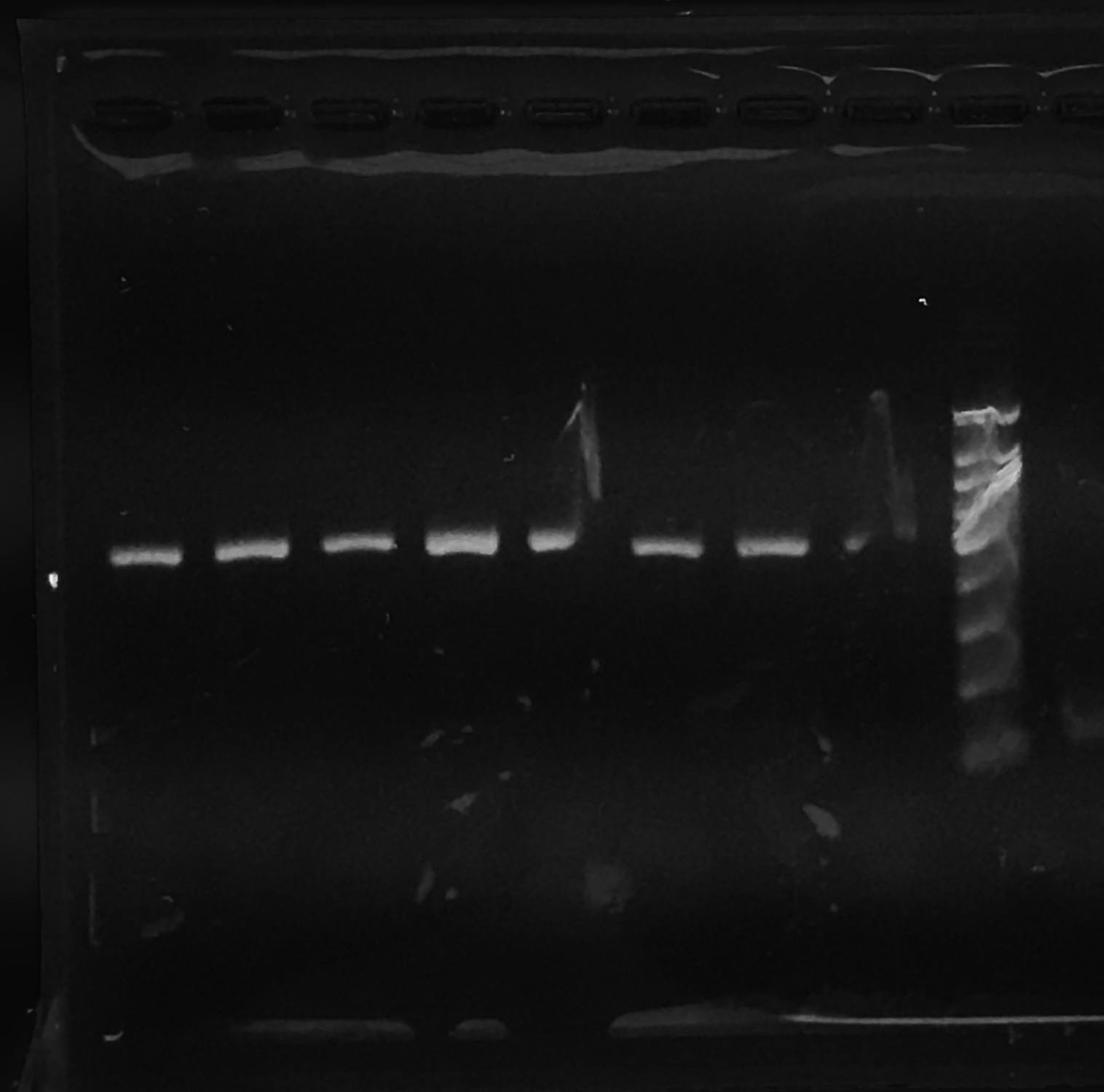

Supplement: Supplementary file 1 [file ijms-19-01178-s001.zip › ijms-284392-supplementary materials/GY59.jpg]

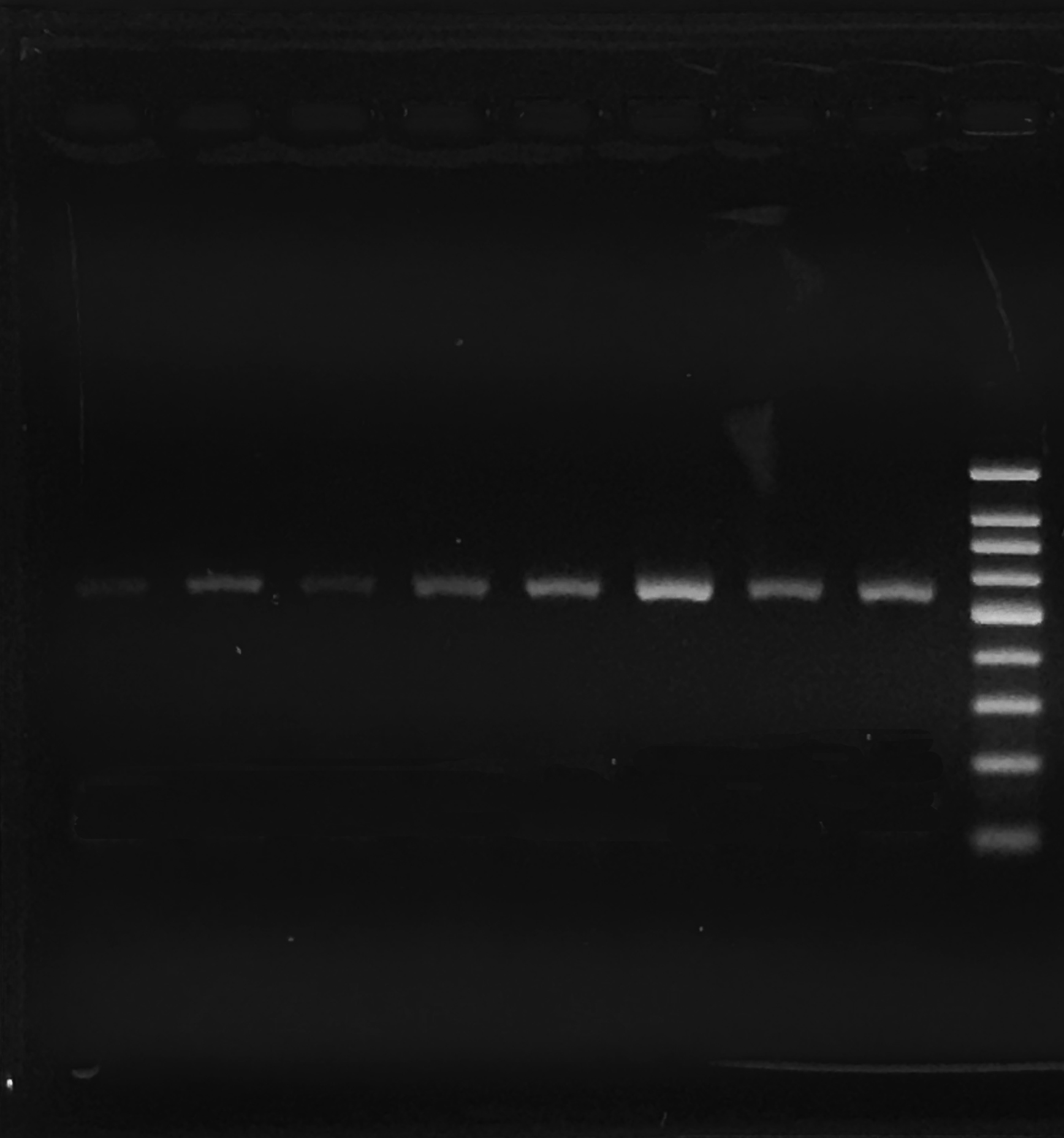

Supplement: Supplementary file 1 [file ijms-19-01178-s001.zip › ijms-284392-supplementary materials/GY60.jpg]

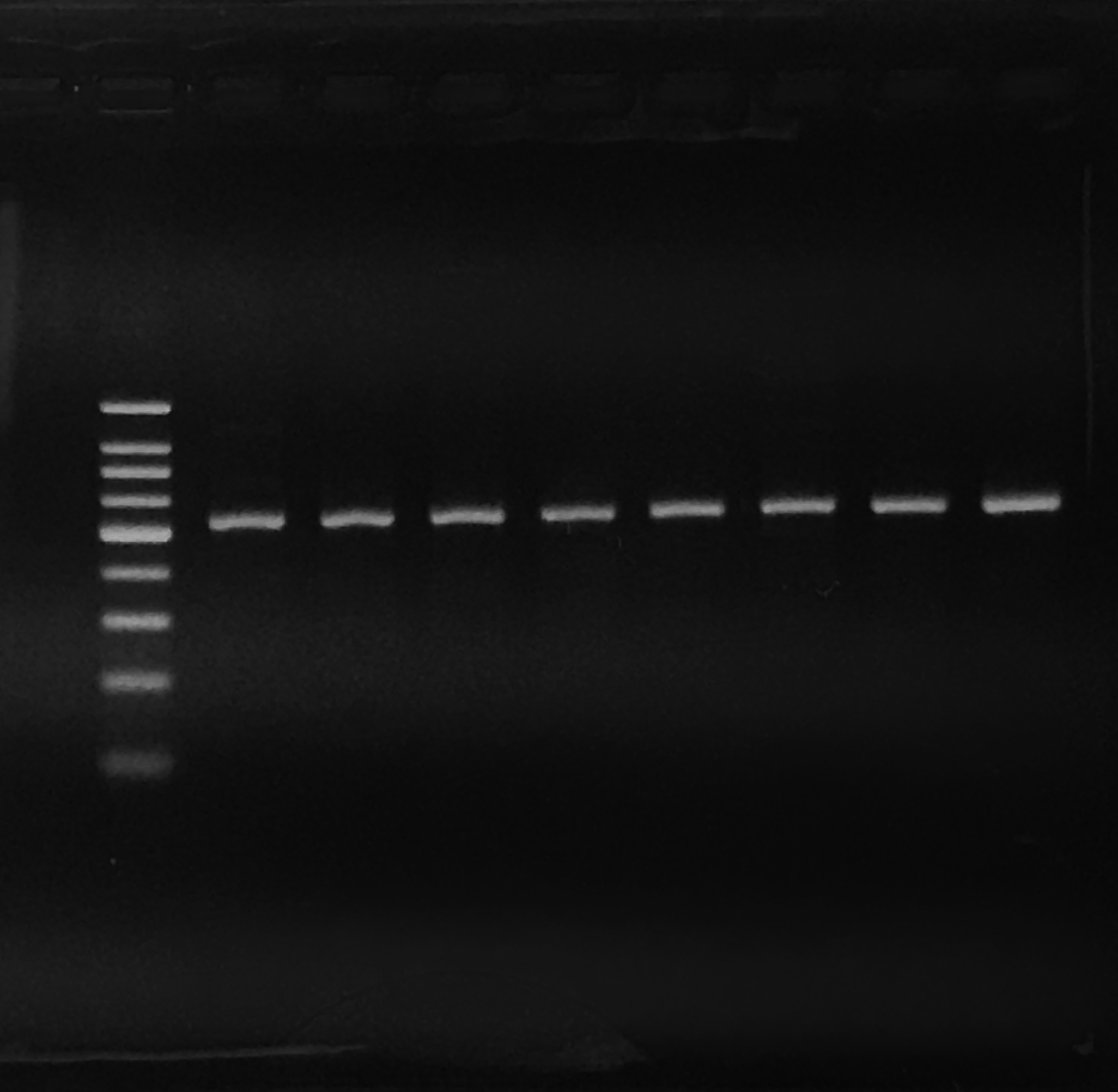

Supplement: Supplementary file 1 [file ijms-19-01178-s001.zip › ijms-284392-supplementary materials/GY66.jpg]

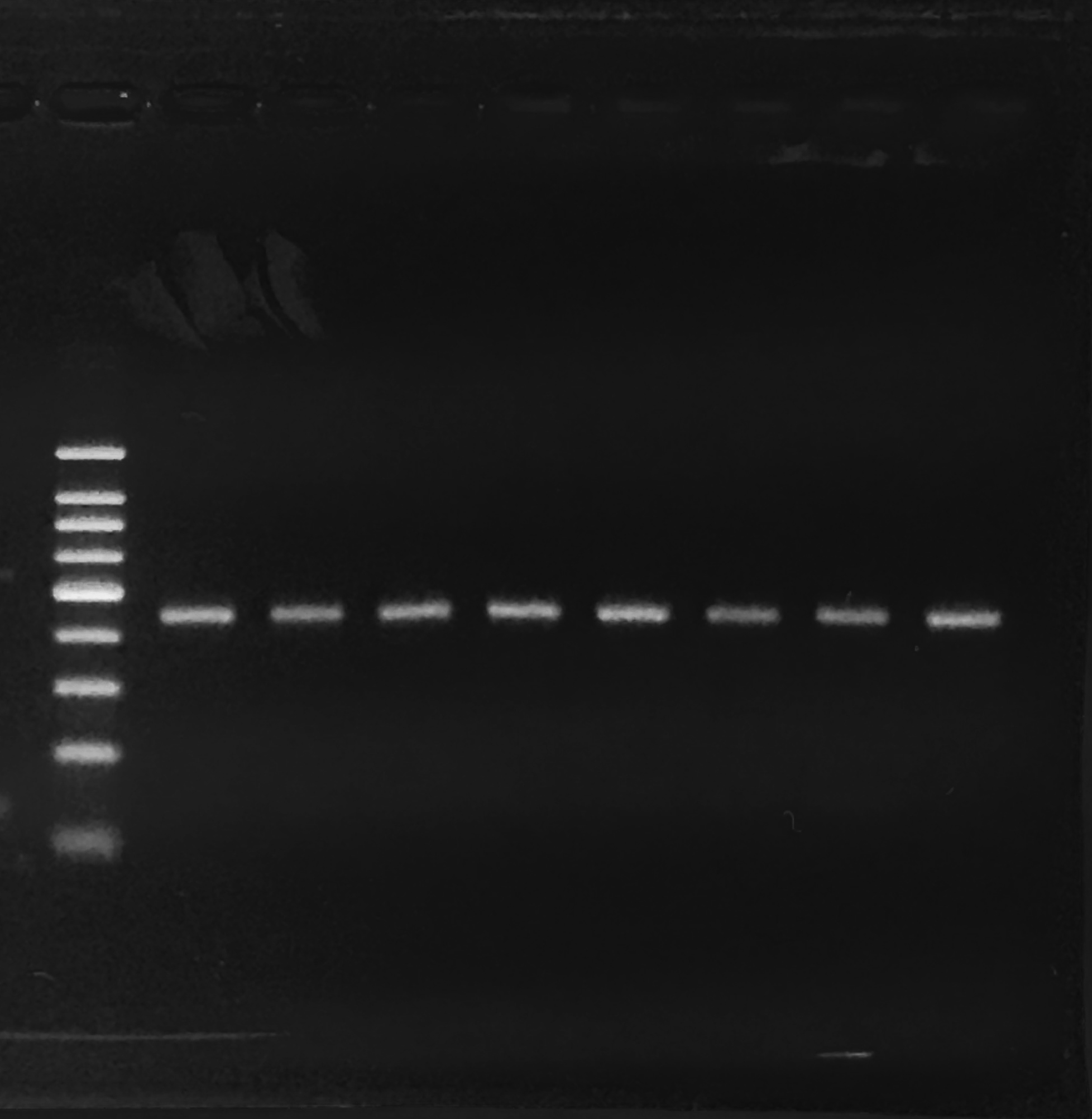

Supplement: Supplementary file 1 [file ijms-19-01178-s001.zip › ijms-284392-supplementary materials/GY67.jpg]

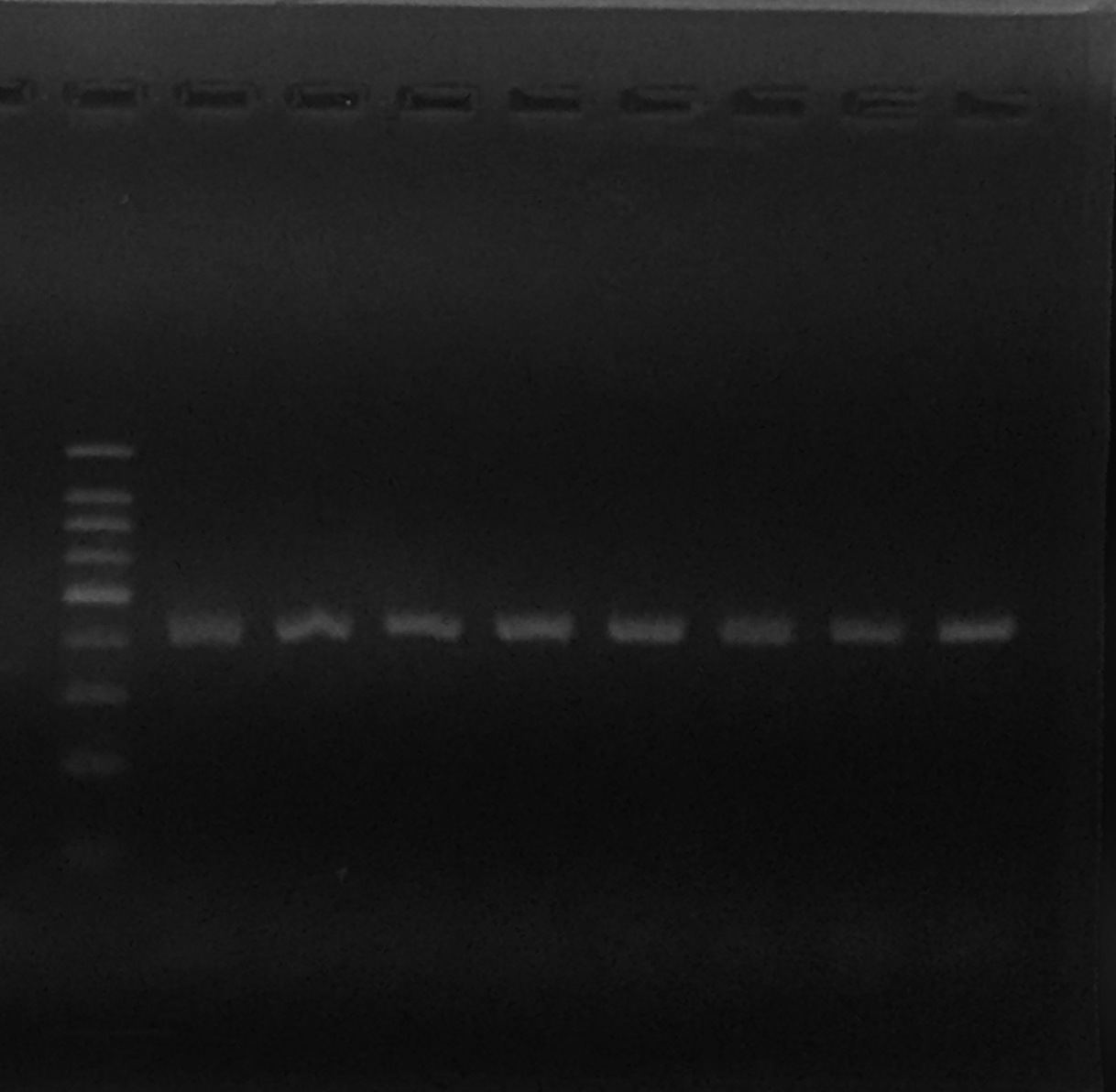

Supplement: Supplementary file 1 [file ijms-19-01178-s001.zip › ijms-284392-supplementary materials/GY68.jpg]

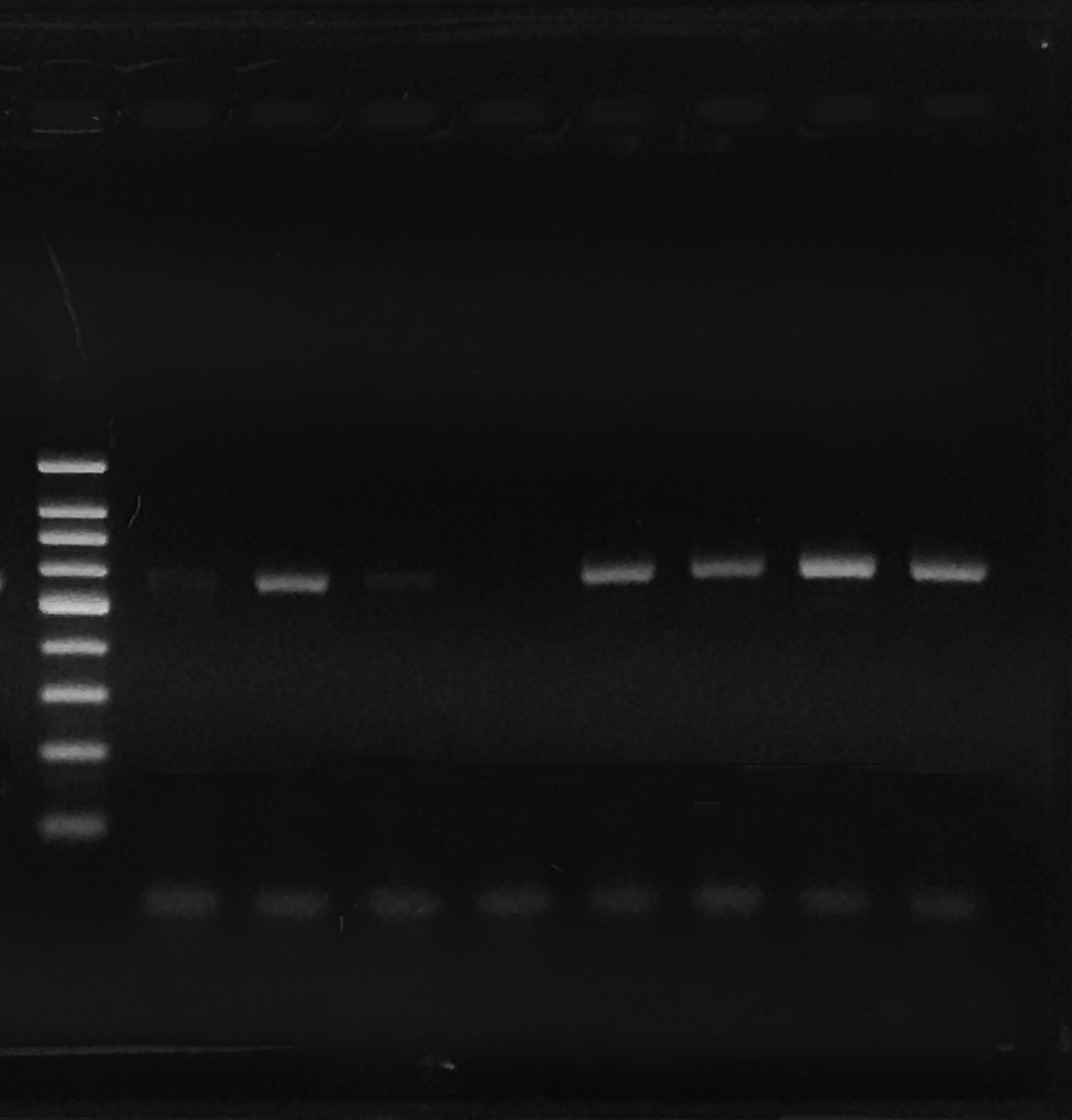

Supplement: Supplementary file 1 [file ijms-19-01178-s001.zip › ijms-284392-supplementary materials/GY69.jpg]

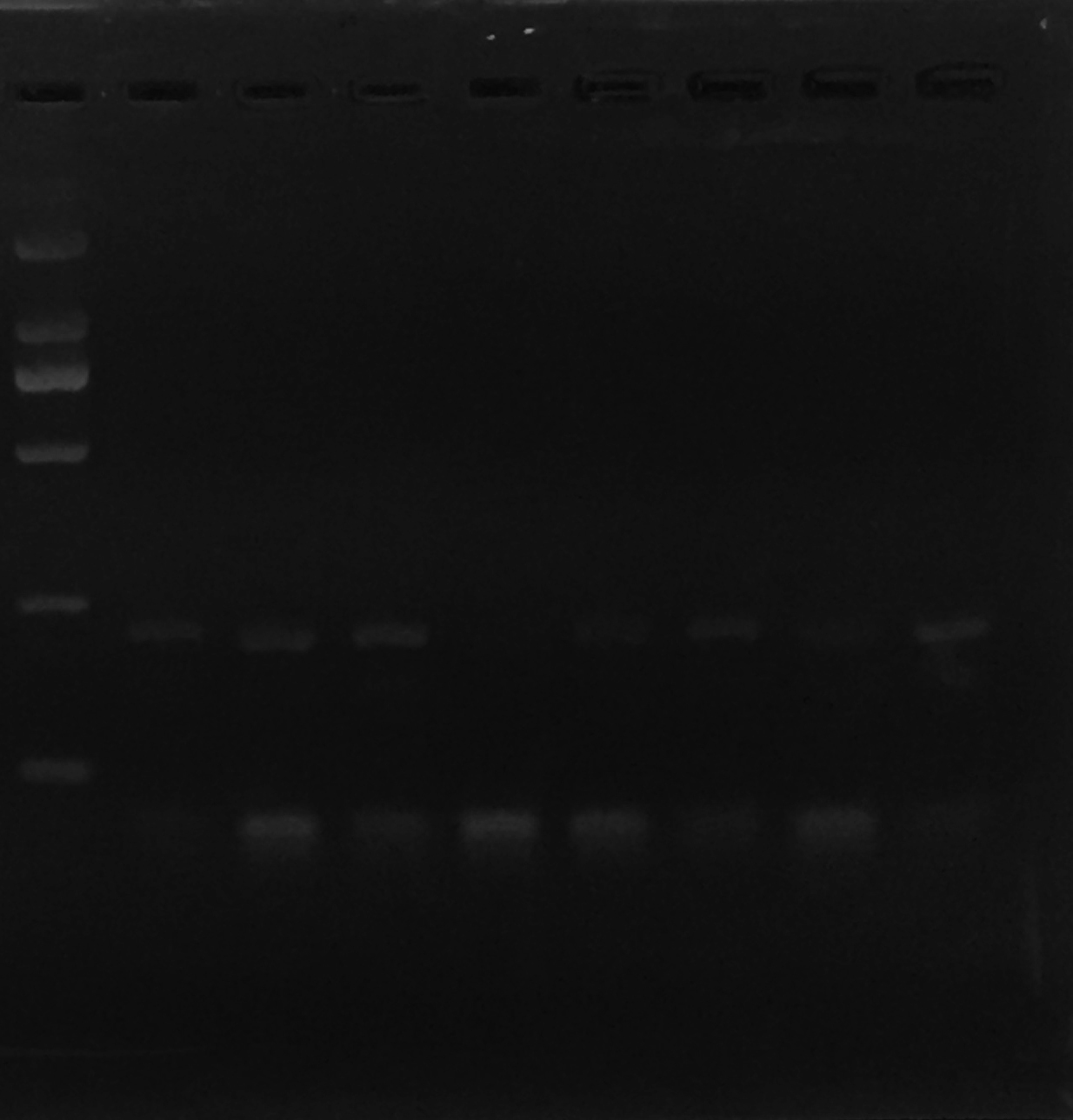

Supplement: Supplementary file 1 [file ijms-19-01178-s001.zip › ijms-284392-supplementary materials/GY7.jpg]

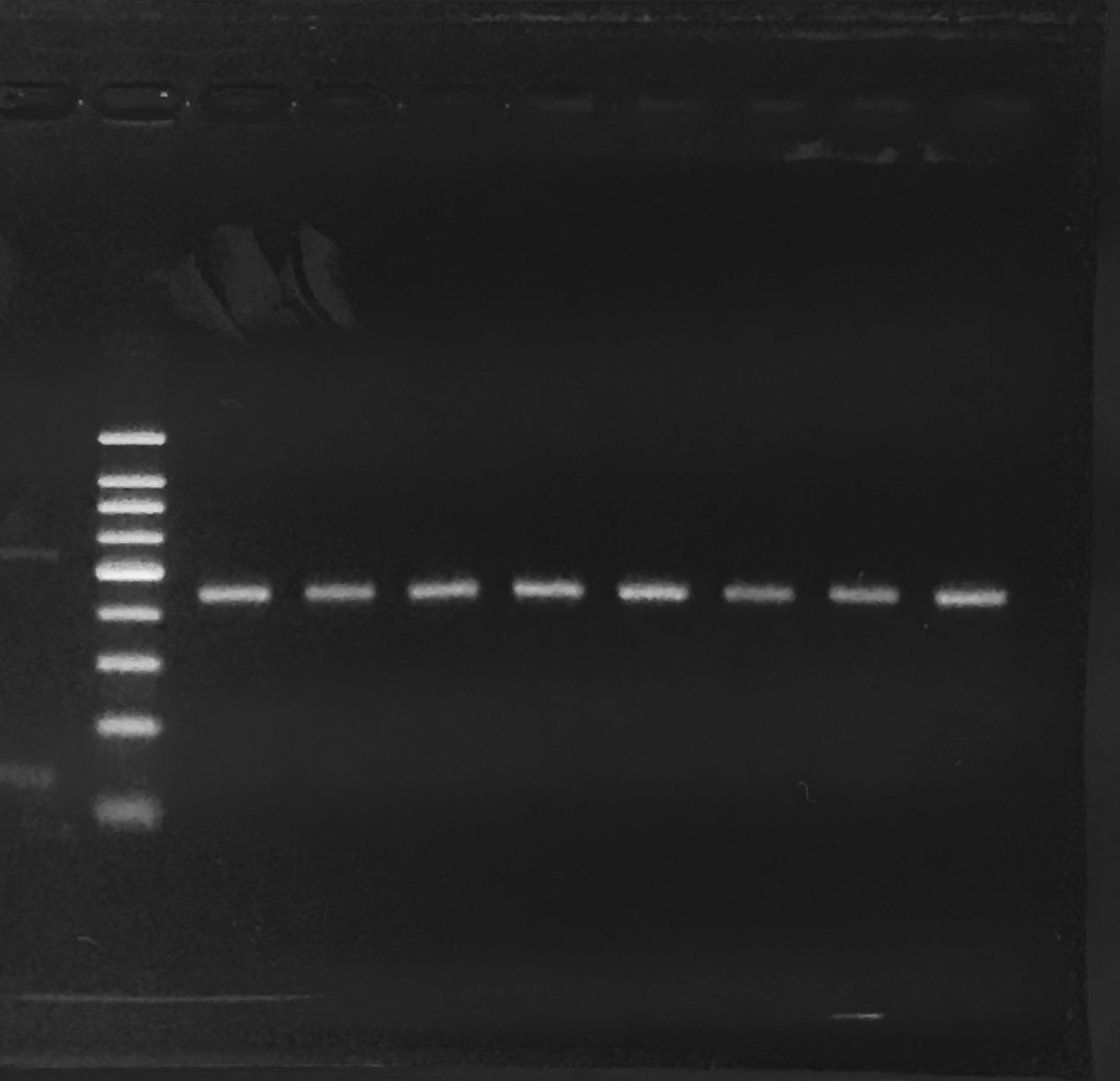

Supplement: Supplementary file 1 [file ijms-19-01178-s001.zip › ijms-284392-supplementary materials/GY70.jpg]

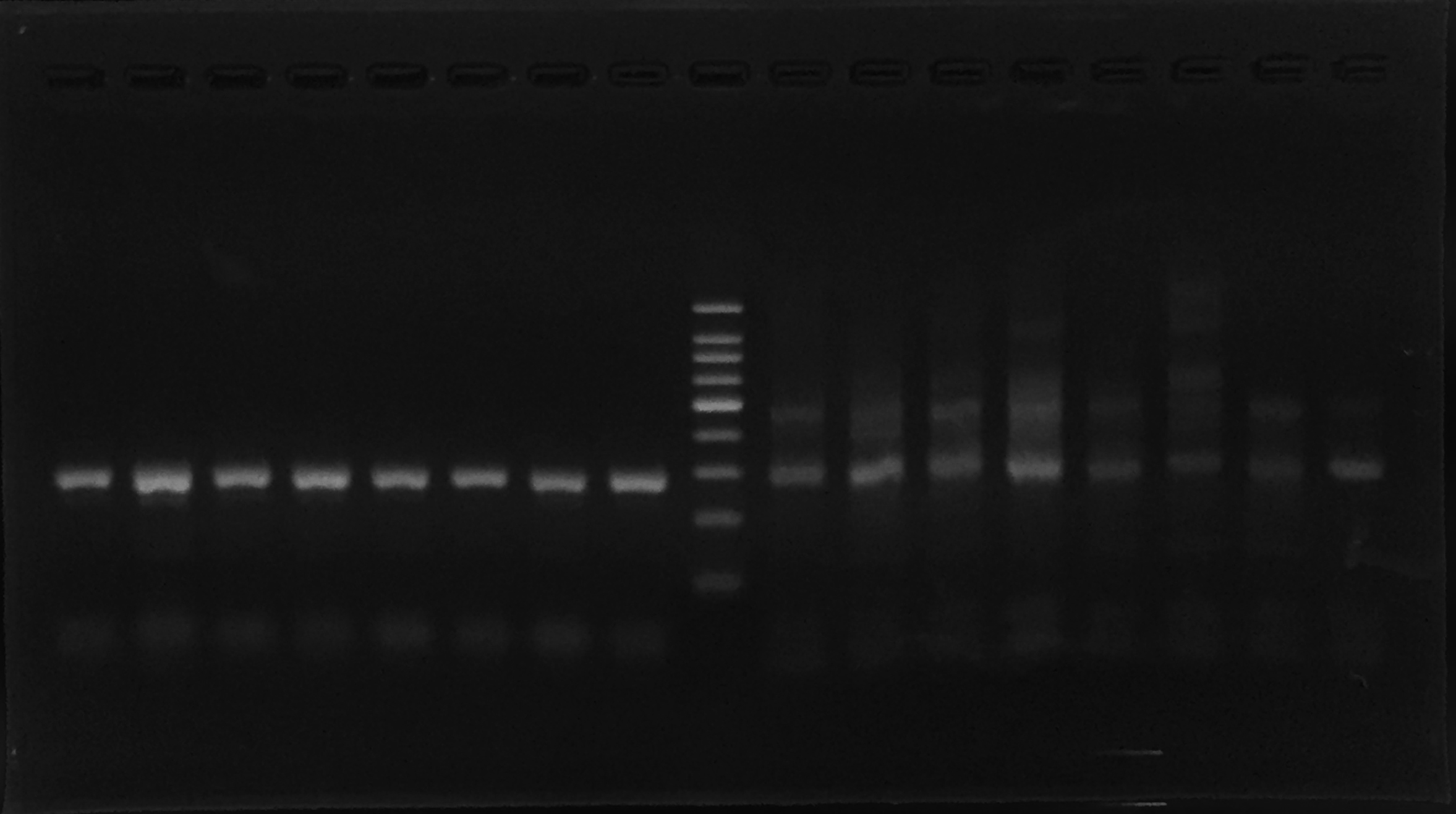

Supplement: Supplementary file 1 [file ijms-19-01178-s001.zip › ijms-284392-supplementary materials/GY71 and GY72.jpg]

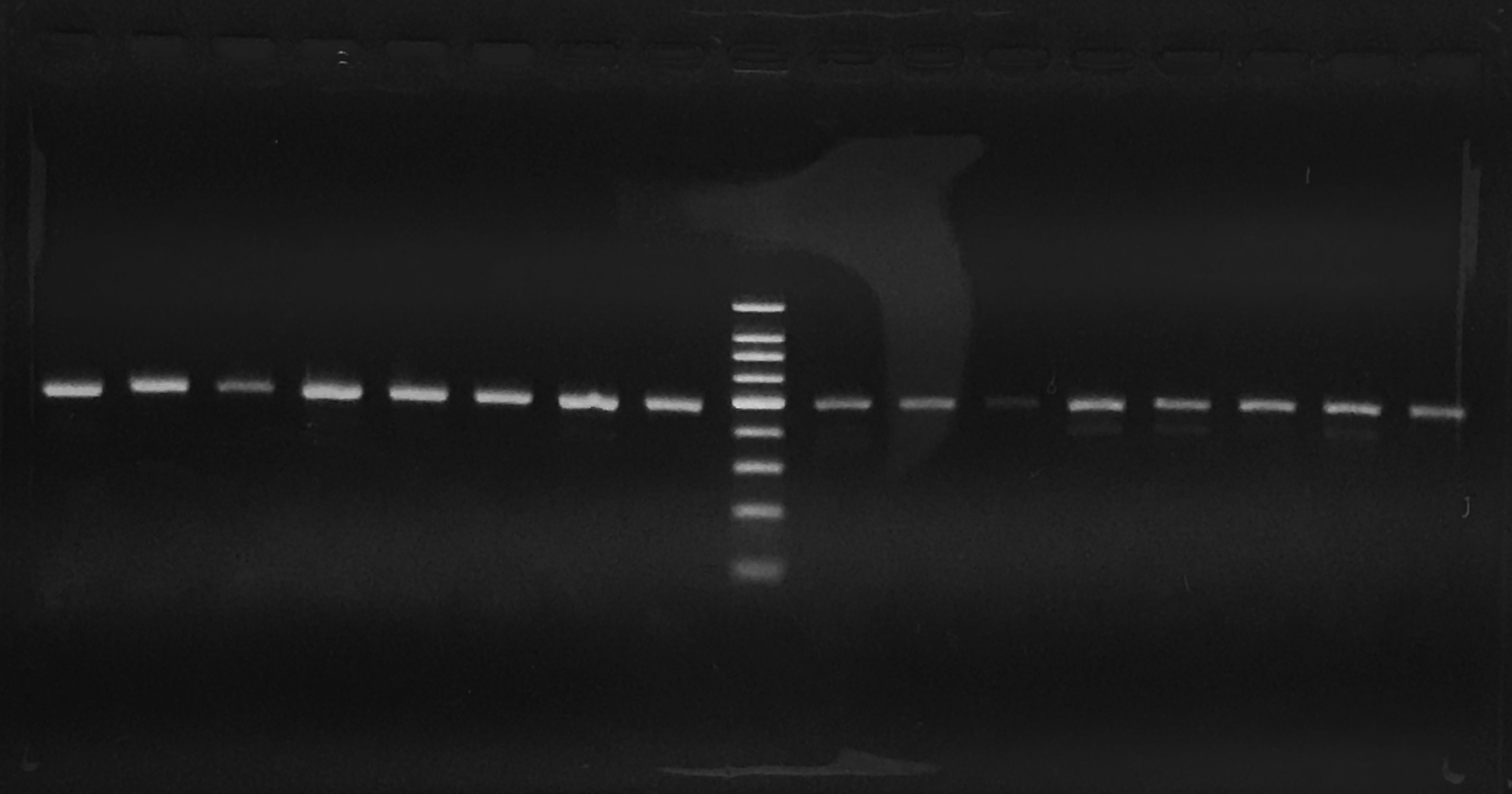

Supplement: Supplementary file 1 [file ijms-19-01178-s001.zip › ijms-284392-supplementary materials/GY74 and GY75.jpg]

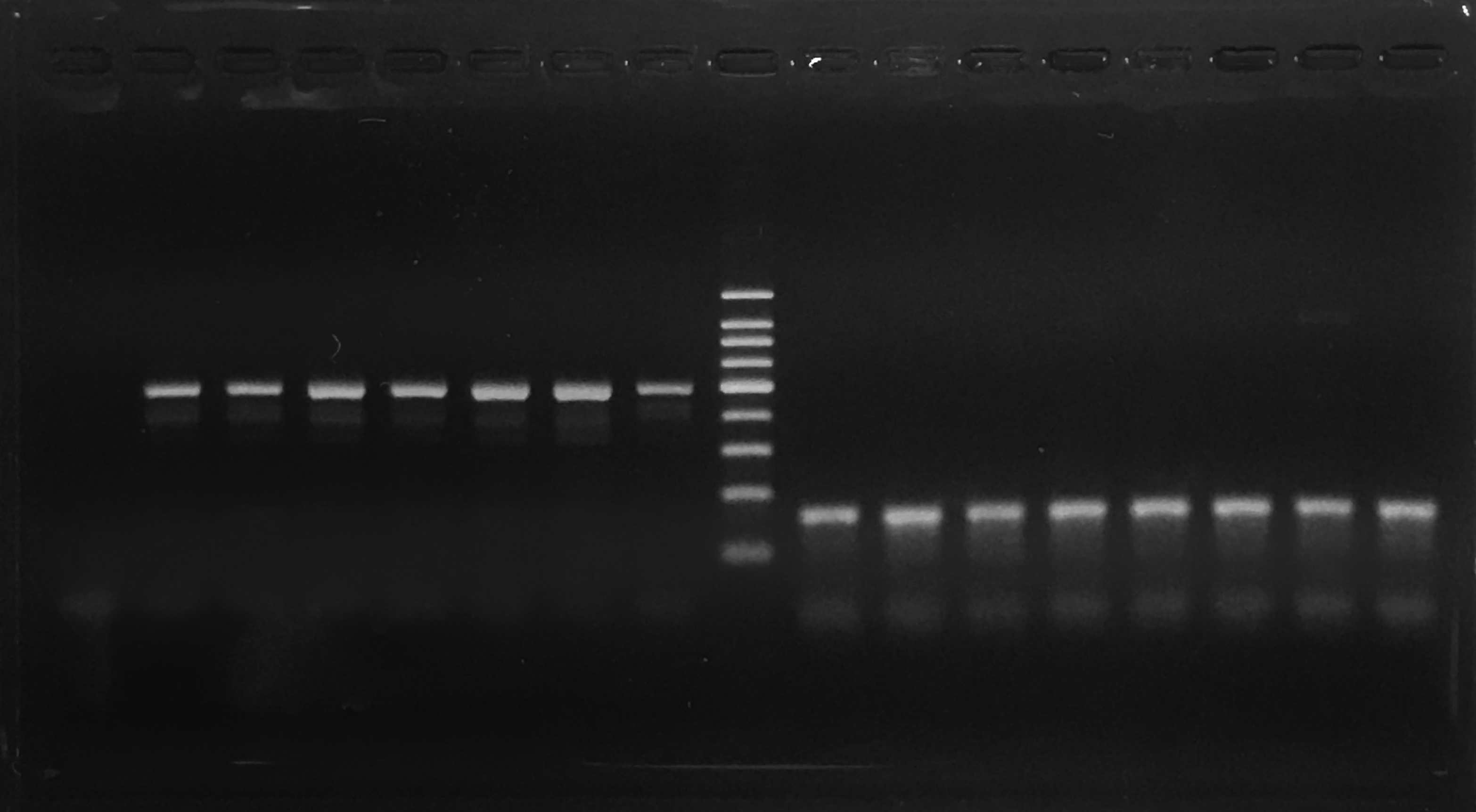

Supplement: Supplementary file 1 [file ijms-19-01178-s001.zip › ijms-284392-supplementary materials/GY76 and GY79.jpg]

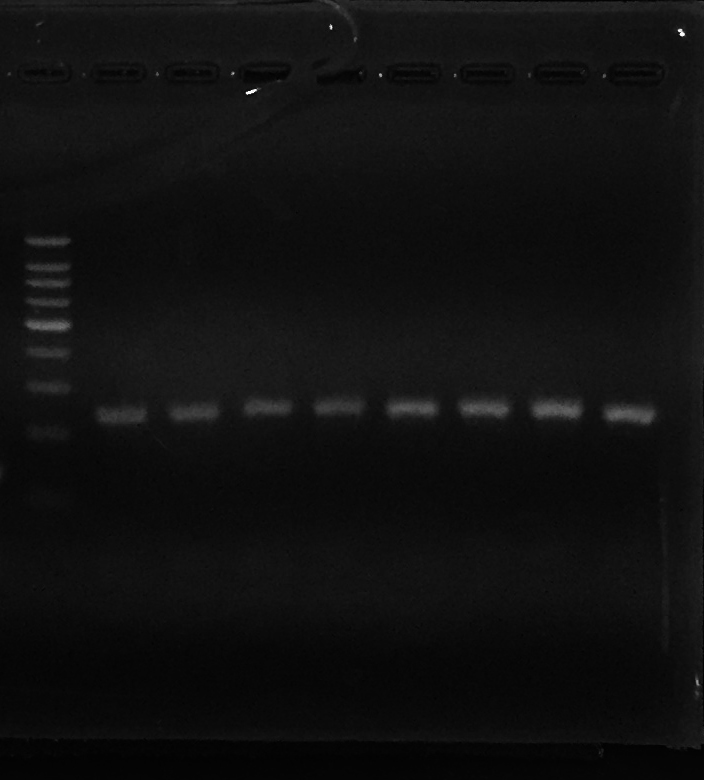

Supplement: Supplementary file 1 [file ijms-19-01178-s001.zip › ijms-284392-supplementary materials/GY81.jpg]

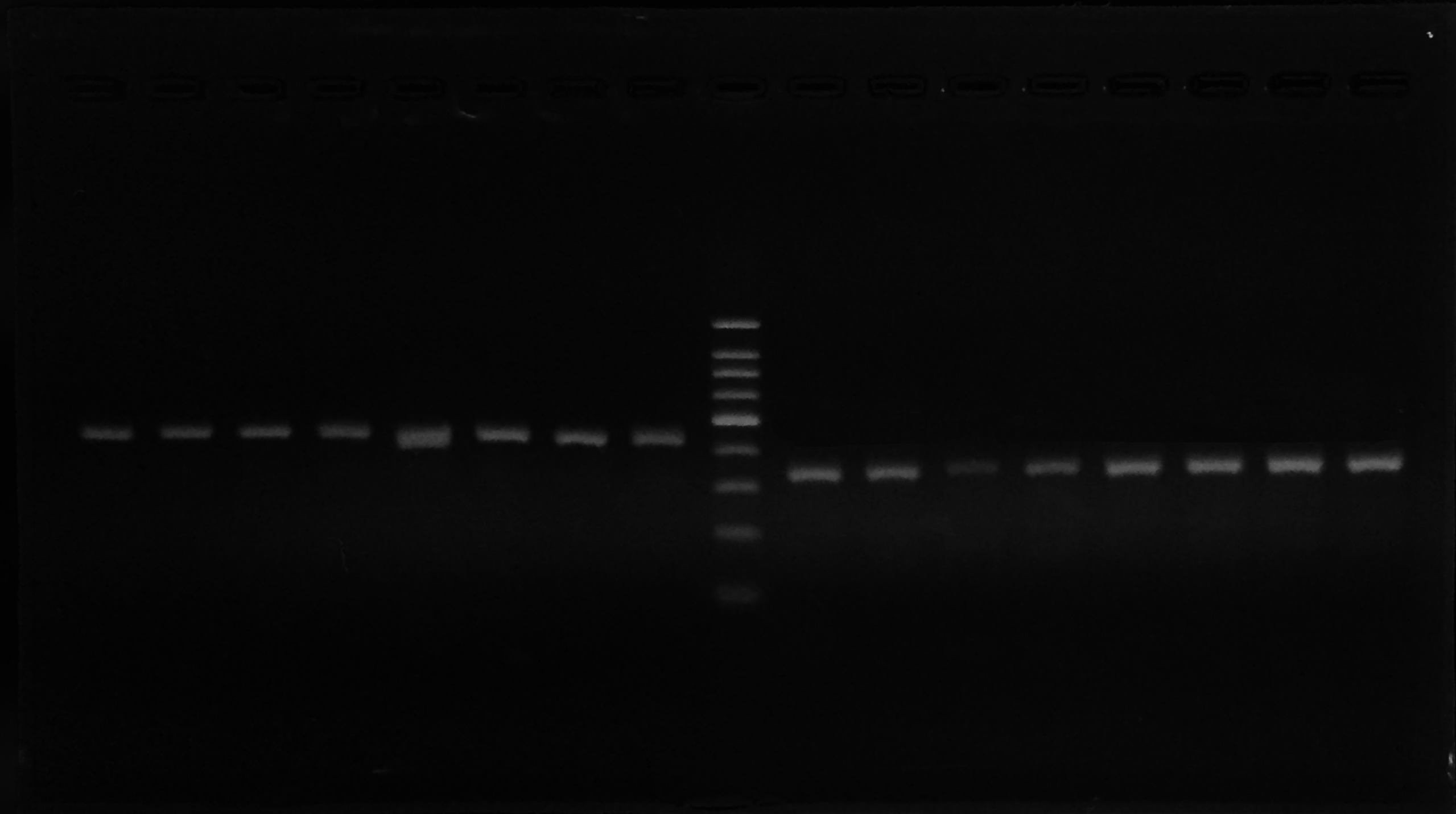

Supplement: Supplementary file 1 [file ijms-19-01178-s001.zip › ijms-284392-supplementary materials/GY86 and GY87.jpg]

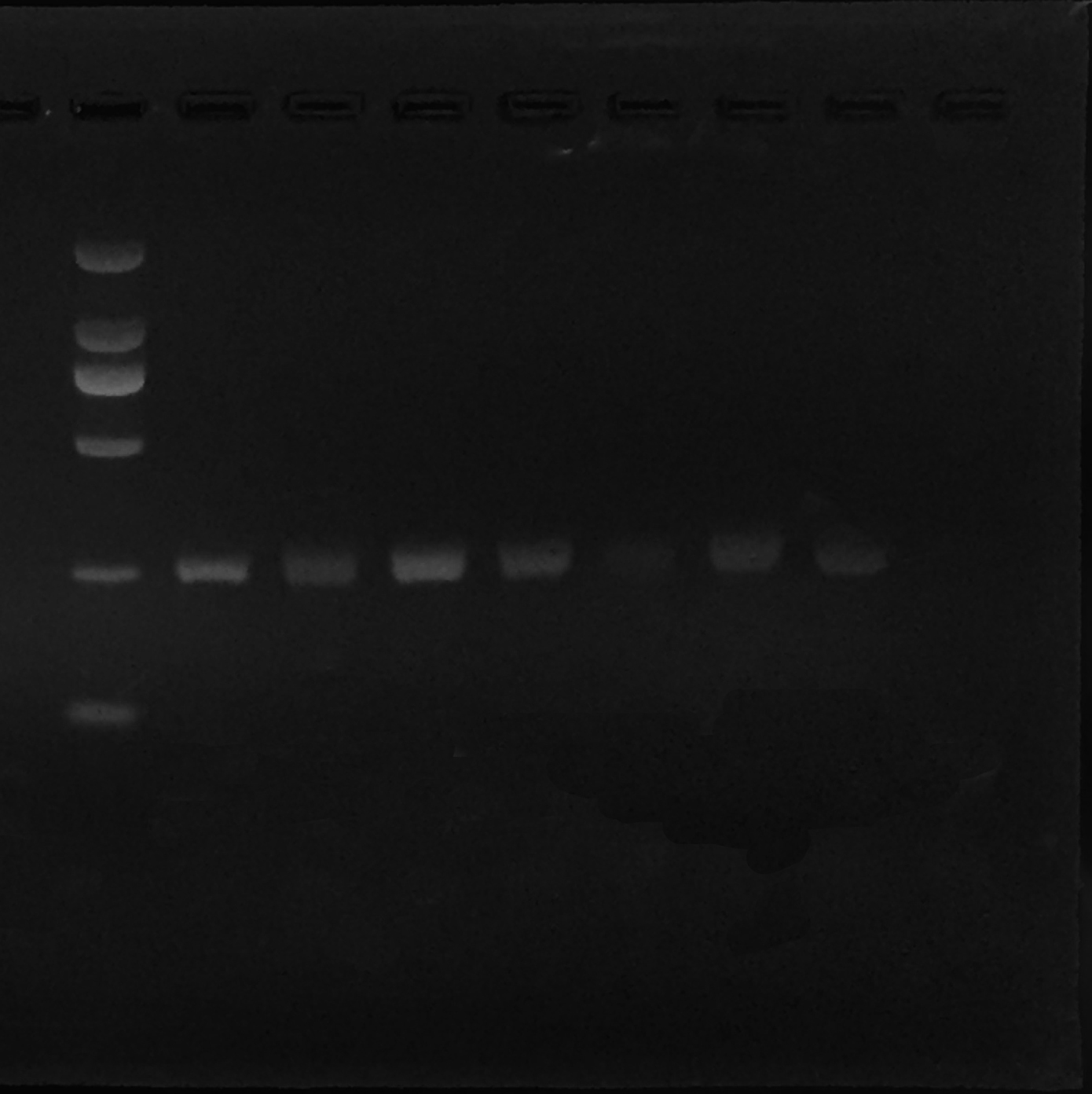

Supplement: Supplementary file 1 [file ijms-19-01178-s001.zip › ijms-284392-supplementary materials/GY9.jpg]
